# Revealing new candidate genes for reproductive traits in pigs: combining Bayesian GWAS and functional pathways

**DOI:** 10.1186/s12711-016-0189-x

**Published:** 2016-02-01

**Authors:** Lucas L. Verardo, Fabyano F. Silva, Marcos S. Lopes, Ole Madsen, John W. M. Bastiaansen, Egbert F. Knol, Mathew Kelly, Luis Varona, Paulo S. Lopes, Simone E. F. Guimarães

**Affiliations:** Department of Animal Science, Universidade Federal de Viçosa, Viçosa, 36570000 Brazil; Animal Breeding and Genomics Centre, Wageningen University, 6700 AH Wageningen, The Netherlands; Topigs Norsvin, Research Center, 6641 SZ Beuningen, The Netherlands; Queensland Alliance for Agriculture and Food Innovation, The University of Queensland, St Lucia, QLD 4072 Australia; Departamento de Anatomía, Embriología y Genética, Universidad de Zaragoza, 50013 Saragossa, Spain

## Abstract

**Background:**

Reproductive traits such as number of stillborn piglets (SB) and number of teats (NT) have been evaluated in many genome-wide association studies (GWAS). Most of these GWAS were performed under the assumption that these traits were normally distributed. However, both SB and NT are discrete (e.g. count) variables. Therefore, it is necessary to test for better fit of other appropriate statistical models based on discrete distributions. In addition, although many GWAS have been performed, the biological meaning of the identified candidate genes, as well as their functional relationships still need to be better understood. Here, we performed and tested a Bayesian treatment of a GWAS model assuming a Poisson distribution for SB and NT in a commercial pig line. To explore the biological role of the genes that underlie SB and NT and identify the most likely candidate genes, we used the most significant single nucleotide polymorphisms (SNPs), to collect related genes and generated gene-transcription factor (TF) networks.

**Results:**

Comparisons of the Poisson and Gaussian distributions showed that the Poisson model was appropriate for SB, while the Gaussian was appropriate for NT. The fitted GWAS models indicated 18 and 65 significant SNPs with one and nine quantitative trait locus (QTL) regions within which 18 and 57 related genes were identified for SB and NT, respectively. Based on the related TF, we selected the most representative TF for each trait and constructed a gene-TF network of gene-gene interactions and identified new candidate genes.

**Conclusions:**

Our comparative analyses showed that the Poisson model presented the best fit for SB. Thus, to increase the accuracy of GWAS, counting models should be considered for this kind of trait. We identified multiple candidate genes (e.g. *PTP4A2, NPHP1,* and *CYP24A1* for SB and *YLPM1, SYNDIG1L, TGFB3,* and *VRTN* for NT) and TF (e.g. *NF*-*κB* and *KLF4* for SB and *SOX9* and *ELF5* for NT), which were consistent with known newborn survival traits (e.g. congenital heart disease in fetuses and kidney diseases and diabetes in the mother) and mammary gland biology (e.g. mammary gland development and body length).

**Electronic supplementary material:**

The online version of this article (doi:10.1186/s12711-016-0189-x) contains supplementary material, which is available to authorized users.

## Background

Reproductive traits, such as number of stillborn piglets (SB) and number of teats (NT), are widely included in the selection indices of pig breeding programs due to their importance to the pig industry. The number of SB is a complex trait that is directly affected by the total number of piglets born [1] and by temporal gene effects in different parities [2]. In humans, it has been shown that kidney diseases and diabetes in the mother and congenital heart disease in the fetus are some of the main causes of the occurrence of stillbirths [3–5]. In pigs, however, a better biological understanding of these traits is still needed to improve selection against SB. Number of teats is a trait with a large influence on the mothering ability of sows [6], since it is a limiting factor for increasing the number of weaned piglets. Biologically, the development of embryonic mammary glands requires the coordination of many signaling pathways to direct cell shape changes, cell movements, and cell–cell interactions that are necessary for proper morphogenesis of mammary glands [7]. In addition, number of vertebrae, which determines the body length of the sow, may also have a direct relation with the final NT that is observed in pigs [8].

Since these traits are directly involved with higher production and welfare of piglets, several genome-wide association studies (GWAS) have been performed for SB and NT [2, 9, 10]. However, in these GWAS, these traits were assumed to be normally distributed, which may not be true. Both SB and NT are measured as count variables, and therefore they follow discrete distributions such as the Poisson distribution. Although the Poisson distribution has already been implemented in animal breeding in the context of traditional mixed models [11, 12] and quantitative trait locus (QTL) mapping [13, 14], there are no reports of GWAS for SB and NT using such models.

Poisson models can be fitted using a Bayesian Markov chain Monte Carlo (MCMC) approach [15]. By applying Poisson models to GWAS with a traditional mixed animal linear model, it is possible to fit all markers simultaneously by using the genomic relationship matrix [16], as performed in genomic best linear unbiased prediction (GBLUP). GBLUP has been widely used in genome-wide selection (GWS), in which the vector of genome enhanced breeding values (GEBV), from each MCMC iteration, can be directly converted into a vector of single nucleotide polymorphism (SNP) allele substitution effects [17, 18]. Based on this, samples of the posterior distribution for each SNP effect are generated at each cycle and significance tests based on highest posterior density (HPD) intervals can be performed [19, 20] in order to identify the most relevant SNPs. In addition, the posterior probability (PPN0) of the estimated effect being smaller than 0 (for negative effects) or greater than 0 (for positive effects), as proposed by Ramírez et al. [20] and Cecchinato et al. [21], can also be used to report the significance of SNP effects under a Bayesian approach. Furthermore, when considering all SNPs simultaneously in the model (in this case, the GBLUP assuming a general shrinkage parameter), some issues such as the influence of gene length (i.e. occurrence of bias that favors genes including larger numbers of SNPs) [22] can be partially minimized by estimating the effect of a particular SNP in the presence of all other SNP effects.

Although many GWAS have been performed, the biological meaning of the identified candidate genes, as well as their functional relationship with transcription factors (TF), still need to be better understood. Results of GWAS can be used for the genetic dissection of complex phenotypes by applying a network approach to the identified genes in the genomic regions around significant SNPs. The genes that are linked to significant SNPs can be used to examine the sharing of pathways and functions, as well as the enrichment of significantly related TF in the selected genes. Some studies have shown that TF genes can be associated with important traits in pigs, e.g. *PIT1* in carcass traits [23] and *SREBF1* in the regulation of muscle fat deposition [24].

Thus, providing evidence for an interaction between a TF gene that is known to be related with a given trait and its predicted target genes via the analysis of regulatory sequences and the construction of gene-TF networks serves as an in silico validation for the gene-gene interactions. A gene-TF network facilitates the identification of the most probable group of candidate genes for the studied trait. In addition, these genes can be used in further functional analyses of in vitro and in vivo validation. Similar approaches have been performed for puberty-related traits in cattle [25, 26] to identify related candidate genes and TF. In pigs, however, this approach has just begun to be exploited [27].

In this study, we performed a Bayesian treatment of a GWAS model assuming Poisson and Gaussian distributions for SB and NT in a commercial pig line. Then, we used the significant SNPs to obtain related genes and generate gene-TF networks, in order to explore the biological roles that underlie the considered traits and identify the most probable candidate genes.

## Methods

### Phenotypic and genotypic data

Stillbirth records from 1390 Large White (LW) sows with an average of 3.9 parities were evaluated. The average SB number in this population was 1.2, ranging from 0 to 16 SB per litter. NT was counted at birth for 1795 LW animals. The average NT in this population was 15.3, ranging from 14 to 20 teats. All animals were genotyped using the Illumina 60 K + SNP Porcine Beadchip [28]. As a part of quality control procedures, SNPs with a GenCall score less than 0.15 [29], a minor allele frequency less than 0.01, a frequency of missing genotypes greater than 0.05, unmapped SNPs, and SNPs located on the Y chromosome according to the Sscrofa10.2 assembly of the reference genome [30] were excluded from the dataset. After quality control, genotypes of 1657 (NT) and 1200 (SB) animals for 41,647 SNPs were included in the association analyses.

### Statistical analyses

Two GBLUP models were fitted to the data under a Bayesian framework. The difference between these models was that one model assumes that the traits follow a Gaussian distribution and the other assumes that the traits follow a Poisson distribution. For the Gaussian response, the following general linear model was assumed:

1 $${\mathbf{y}} = {\mathbf{X }}{\boldsymbol{\upbeta}} + {\mathbf{Zu}} + {\mathbf{Wp}} + {\mathbf{e}} ,$$

where **y** is the vector of phenotypic observations, **β** is the vector of systematic environmental effects, **u** is a vector of additive genetic effects, **p** is a vector of permanent environment effects (fitted only in the model for SB), **e** is a vector of residual effects, and **X**, **Z,** and **W** are design matrices related to **β**, **u,** and **p**, respectively. Herd-year-season (HYS) was considered as a systematic effect for both SB and NT, while a sow parity number was used only for SB and a sex effect was used only for NT. Sex effects are commonly accounted for when analyzing NT according to Lopes et al. [31], who showed that males had on average 0.35 ± 0.09 more teats than females.

For the Poisson response **y**, a latent variable (**l**) was introduced by means of the canonical parameter **λ** (often called the rate or mean parameter) of the Poisson distribution and the link function on the log scale, i.e., y_i_ ∼ Poi(λ = exp (**l**_i_)), where exp is the inverse link function. In this case, the linear model presented in (1) can be applied to the latent variable as follows:

2 $${\mathbf{l}} = {\mathbf{X }}{\boldsymbol{\upbeta}} + {\mathbf{Zu}} + {\mathbf{Wp}} + {\mathbf{e}} .$$

Under a traditional Poisson mixed model [32], the conditional mean and variance of observations must be identical but the absence of some explanatory factors and sources of variation may result in a discrepancy (over-dispersion) between these quantities. The extra residual (**e**) term in Model (2) absorbs all unaccounted sources of variation, while the traditional generalized linear model has no term to which this extra variance can be allocated [33]. Model (2) was initially proposed by Tempelman and Gianola [33], who assumed a gamma distribution for the latent variable in a conjugate conditional distribution from which the parameter estimates were obtained by using a maximum a posteriori method via the Newton–Raphson algorithm. Since the computational simplicity of MCMC methods makes it possible to extend the distributions of the latent variable, we opted for the Gaussian distribution instead of a gamma distribution in order to directly estimate the residual variance, which is defined by functions of the parameters of the gamma distribution under the Tempelman and Gianola [33] approach. Implementation of MCMC algorithms (Gibbs sampler and Metropolis–Hastings) through the conditional posterior distributions for parameters of Model (2), and consequently for Model (1) by replacing **l** by **y,** are presented in detail on pages 36 and 37 of Sun et al. [34].

In the Bayesian analysis, the following prior distributions were assumed for the parameters of Models (1) and (2): **β** ∼ N(0, σ_β_^2^**l**), where σ_β_^2^ is known and assumed to be large (in this case 1e + 10) in order to represent vague prior knowledge; **u** ∼ N(0, σ_u_^2^**G**) and p ∼ N(0, σ_p_^2^**I**), with **I** as an identity matrix and **G** the genomic relationship matrix (covariances between individuals based on observed similarity at the genomic level) proposed by Van Raden [16]. Thus, ${\mathbf{G}} = \frac{{{\mathbf{MM}}^{\varvec{'}} }}{{2\mathop \sum \nolimits_{{{\text{i}} = 1}}^{\text{N}} {\text{p}}_{\text{i}} (1 - {\text{p}}_{\text{i}} )}} ,$ where **M** is the SNP genotype matrix, p_i_ is the allele frequency at SNP i, N is the number of SNPs, and the sum is over all SNPs. This matrix was accessed from the *kin* function from the *synbreed* package in R software, which requires the values 0, 1, and 2 for genotypes aa, aA, and AA, respectively.

For the variance components (σ_u_^2^, σ_p_^2^ and σ_e_^2^), an inverted Chi squared distribution was considered: σ_u_^2^|V_u_, $${\text{S}}_{\text{u}} \sim {\text{V}}_{\text{u}} {\text{S}}_{\text{u}} {\text{X}}_{{{\text{v}}_{\text{u}} }}^{ - 2} ,$$$$\upsigma_{\text{p}}^{\text{2}} \text{|}{\text{V}}_{\text{p}} ,$$$${\text{S}}_{\text{p}} \sim {\text{V}}_{\text{p}} {\text{S}}_{\text{p}} {\text{X}}_{{{\text{v}}_{\text{p}} }}^{ - 2} ,$$ and $${{\upsigma }}_{\text{e}}^{2} | {\text{V}}_{\text{e}} ,$$$${\text{S}}_{\text{e}} \sim {\text{V}}_{\text{e}} {\text{S}}_{\text{e}} {\text{X}}_{{{\text{v}}_{\text{e}} }}^{ - 2} ,$$ where V (degree of freedom) and S (scale parameter) are hyperparameters. Assuming that $${{\upsigma }}^{2} \sim {\text{Scale X}}^{ - 2} ({\text{V}}, {\text{S}}) ,$$ where S = Vσ^2*^ and σ^2*^ is the prior most likely value of σ^2^ [35], the mode of this distribution was equalized to σ^2*^ in order to set the hyperparameter V, i.e. σ^2*^ = VS/(V + 2) = VVσ^2*^/(V + 2). Thus, we have V^2^−V−2 = 0, where the roots are V = −1 and V = 2. Since by definition V > 0, we opted for V = 2 and S = Vσ^2*^, where σ^2*^ was assessed by the variance component estimates from other studies of this same commercial population for SB [36, 37] and NT [31, 38]. To calculate S, the values assumed for $${{\upsigma }}_{\text{u}}^{{2^{ *} }}$$, $${{\upsigma }}_{\text{p}}^{{2^{ *} }}$$ and $${{\upsigma }}_{\text{e}}^{{2^{ *} }}$$ were, respectively, 0.3575, 0.33 and 3.9125 for SB, and 0.43, 0.0221 and 0.71 for NT.

In both Models (1 and 2), the residual vector was assumed to be ${\mathbf{e}}\sim {\text{N}}(0, {{\upsigma }}_{\text{e}}^{2} {\mathbf{I}})$, thereby implying a Gaussian likelihood function. In agreement with Hadfield and Nakagawa [15], the conditional probability of the latent variable is proportional to the product of two terms, the Poisson likelihood of the data given **l** and the Gaussian likelihood based on residual terms, i.e., P(**l**_i_|**y**, **β**, **u**, **p**, σ_u_^2^, σ_p_^2^, σ_e_^2^) ∝ ∏_i=1_^N^P_i_(y_i_|**l**_i_)P(e_i_|σ_e_^2^). Thus, in Model (2), the vector of latent variables (**l**) was also considered to be an unknown parameter, therefore not allowing for a fully recognizable conditional distribution and requiring the use of the Metropolis–Hastings algorithm to generate samples of posterior distribution for **l**. For the other unknown parameters $({\varvec{\upbeta}}, {\mathbf{u}}, {\mathbf{p}},{{\upsigma }}_{\text{u}}^{2} ,{{\upsigma }}_{\text{p}}^{2} , {{\upsigma }}_{\text{e}}^{2} )$, given the closed form of the full conditional posterior distributions, the Gibbs sampler algorithm was used. For the standard linear mixed Model (1) with a Gaussian response and identity link, P(**l**_i_|**y**, **β**, **u**, **p**, σ_u_^2^, σ_p_^2^, σ_e_^2^) is always unity, and so the Metropolis–Hastings steps are not required.

Models (1) and (2) were fitted to the data using an adaptation of the MCMCglmm R package [39], with a total of 100,000 iterations, while assuming a burn-in period of 50,000 and a sampling interval (thin) each second iteration. The adaptation involved the use the inverse of the **G** matrix instead of the inverse of the traditional relationship matrix (**A**) in the *ginverse* option of this package.

To evaluate convergence of the MCMC chains, we used the *boa.geweke* function from the *boa* (Bayesian Output Analysis) package in the R software. This function enables users to define a matrix in which columns and rows contain the monitored parameters and the MCMC iterations, respectively. The output directly provides the Geweke Z-Scores and associated p values for each one of these parameters, thus allowing for the use of descriptive statistics to make inferences about convergence in the presence of a large number of parameters (marker effects and breeding values), as considered here. Descriptive statistics and histograms for p values from the Geweke test for all parameters, as well as trace plots for the most significant SNPs, are in Additional file 1: Figure S1, Additional file 2: Figure S2, and Additional file 3: Figure S3.

Models (1) and (2) were compared based on the deviance information criterion (DIC) developed by Spiegelhalter et al. [40]: ${\text{DIC}} = {\text{D}}\left(\bar{\uptheta} \right) + 2{\text{p}}_{\text{D}} ,$ where ${\text{D}}\left(\bar{\uptheta} \right)$ is a point estimate of the deviance that is obtained by replacing the parameters with their posterior mean estimates in the log-likelihood function, i.e. ${\text{D}}\left(\bar{\uptheta} \right) = - 2\log [{\text{P}}({\mathbf{y}}|{\hat{\boldsymbol{\upbeta }}},{\hat{\mathbf{u}}}, {\hat{\mathbf{p}}},{\hat{{\upsigma }}}_{\text{u}}^{2} ,{\hat{{\upsigma }}}_{\text{p}}^{2} ,{\hat{{\upsigma }}}_{\text{e}}^{2} )]$ and ${\text{D}}\left(\bar{\uptheta}\right) = - 2\log [{\text{P}}({\mathbf{l}}|{\mathbf{y}},{\hat{\boldsymbol{\upbeta }}},{\hat{\mathbf{u}}}, {\hat{\mathbf{p}}},{\hat{{\upsigma }}}_{\text{u}}^{2} ,{\hat{{\sigma }}}_{\text{p}}^{2} ,{\hat{{\upsigma }}}_{\text{e}}^{2} )] ,$ respectively, for the Gaussian and Poisson models, and is the effective number of parameters. Models with a smaller DIC are preferred to models with a larger DIC.

One important statistical feature of the present study was the fact that the vector ${\hat{\mathbf{u}}}$ (GEBV) was kept at each kth MCMC iteration (${\hat{\mathbf{u}}}^{{\text{(k)}}}$) for Models (1) and (2), which enabled us to generate the vector of SNP effects (**α**) for each iteration using the following linear system: ${\hat{\boldsymbol{\upalpha }}}^{{({\text{k}})}} = {\mathbf{M}}'({\mathbf{MM}}')^{ - 1} {\hat{\mathbf{u}}}^{{({\text{k}})}}$ [18], where (**MM**′)^−1^ is the generalized inverse and **M** is the incidence matrix for SNP effects based on the SNP genotypes used in **G** matrix definition, as previously presented. Once a vector of SNP effects was generated for each iteration (${\hat{\boldsymbol{\upalpha }}}^{{({\text{k}})}}$), it was possible to obtain a MCMC chain of 25,000 iterations (see burn-in and thin previously mentioned) for each SNP. Thus, after verifying convergence of these chains by the Geweke and Raftery and Lewis criteria (using, respectively, the functions *geweke.diag* and *raftery.diag* of coda R package) [41], a sample of the posterior distribution for the effect of each SNP was obtained. These distributions allowed the calculation of the 95 % HPD (highest posterior density) intervals, as presented by Li et al. [19], and determination of the posterior probability (PPN0) that the SNP effect is greater (for positive effects) or smaller than (for negative effects) zero, as presented by Ramírez et al. [20] and Cecchinato et al. [21]. The HPD intervals and PPN0 were obtained for each SNP and the chromosomal positions of the significant SNPs were used to identify genes that influence the target traits.

Obtaining the SNP effect directly from **M**′(**MM**′)^−1^ and storage of the chains for the effect of each SNP required the use of a large amount of data, which was facilitated by using the Intel Math Kernel Library, which is highly optimized for use on Intel processers and uses a parallel process to decrease computational time. The application of these optimized libraries for matrix computation in genomic issues was discussed in detail in Aguilar et al. [42]. The computer that was used to perform the analyses had 12 cores running Intel^(R)^ Core^(^™^)^ i7-3930 K CPU @ 3.20 GHz and 96 gb of ram.

Linkage disequilibrium (LD) between significant SNPs was evaluated using Haploview [43] to identify QTL regions on chromosomes, using the default parameters based on Gabriel et al. [44]. 95 % confidence bounds on D′ [45] were used to determine if a pair of SNPs was in “strong LD”.

### Gene-TF networks

Genes that overlapped with significant QTL regions and with individual significant SNPs, along with, for both, a 32.5 kb flanking sequence (half the average distance between SNPs present on the chip), were identified at the dbSNP NCBI web site (http://www.ncbi.nlm.nih.gov/SNP/). When checking these segments, we identified the presence of any gene that could be related with a QTL or a significant SNP. In addition, studies on the Large White pig breed have demonstrated that even for an average distance between two SNPs of around 200–250 kb, the LD (r^2^) is still high (0.31), with an average LD greater than 0.2 reported to be necessary for genomic analyses [46, 47]. The identified overlapping genes were then used to obtain functional gene ontology (GO) terms and pathways with GeneCards (http://www.genecards.org/) and TOPPCLUSTER (http://toppcluster.cchmc.org/).

TF enriched in the identified set of genes were found with the TFM-Explorer program (http://bioinfo.lifl.fr/TFM/TFME/). This program uses weight matrices from the JASPAR database [48] to detect all potential TF binding sites (TFBS) from a set of gene sequences and searches for locally overrepresented TFBS. Then, it gives significant clusters (TFBS regions of the input sequences associated with a factor) by calculating a score function with a threshold (*p* value) equal or greater than 10^−3^ for each position and each sequence, such as described in Touzet and Varré [49]. The program’s default size for the analyzed promoter region is 2000–200 bp. However, annotations of the genes’ transcription start sites (TSS) are uncertain for some regions in the current assembly, thus to compensate for inaccurate annotated TSS, both in the 5′ and 3′ direction, we increased (no restriction) the limits of the promoter regions, i.e. for the set of genes for each trait, excluding *ncRNA* genes, we collected 3000 bp upstream and 300 bp downstream sequences (FASTA format) of the genes’ TSS, based on the Sscrofa10.2 assembly at the NCBI web site. This data was then used as input for TFM-explorer and the given list of TF was fed into Cytoscape [50] using a Biological Networks Gene Ontology tool (BiNGO) plugin [51] to identify significantly overrepresented GO terms. Based on overrepresented biological processes in BiNGO and evidence from a review of the literature, we were then able to identify the most representative TF (according to their biological role and literature evidence) that were related to SB and NT to construct a network with gene-TF interactions. With the goal of identifying the most likely candidate genes for the studied traits SB and NT, we applied the Network Analyses Cytoscape tool for number of TFBS and consequently, number of connections in each gene, to determine those that were most strongly connected with SB and NT. Thus, genes with the higher TFBS for the most representative TF were highlighted on the gene-TF network.

## Results

### Statistical analyses

The convergence diagnostics of MCMC chains for all estimated parameters are summarized in Additional file 1: Figure S1, Additional file 2: Figure S2, and Additional file 3: Figure S3. All MCMC chains achieved the convergence according to Geweke’s test. There was no evidence that allowed us to reject the null hypothesis (stationarity of the MCMC chains) under a significance level of 5 %. This result is illustrated by histograms and descriptive statistics (mean, median, standard deviation, minimum and maximum) for the p values from Geweke’s test.

**Fig. 1:**
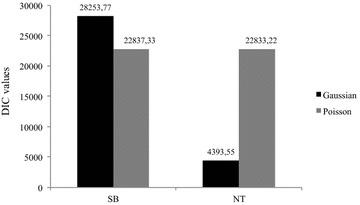
Deviance information criterion (DIC) plot. DIC values obtained using Gaussian (*black*) and Poisson (*gray*) models for stillborn (SB) and number of teats (NT) data

The most appropriate model (Gaussian or Poisson) was determined for each trait (SB and NT) by using DIC (Fig. 1). For SB, the DIC was 5416.44 times smaller with the Poisson model than with the Gaussian model. In contrast, for NT, the Gaussian model had a DIC that was 18439.67 times smaller than with the Poisson model. According to Spiegelhalter et al. [40], models with differences in DIC less than 2 must be equally considered, while models that have DIC that are between 2 and 7 have considerably less support. Thus, by using these differences in DIC as a reference, the Poisson model was clearly superior for SB and the Gaussian was clearly superior for NT. Histograms for the original phenotypes when considering the fit of the Poisson and Gaussian distributions are in Additional file 4: Figure S4. Thus, the remainder of the analyses presented here for SB and NT used the Poisson and Gaussian models, respectively.

Using these distributions, we identified 18 SNPs related to SB and 65 to NT [see Additional files 5: Table S1, Additional file 6: Table S2, Additional file 7: Figure S5 and Additional file 8: Figure S6]. Significant SNPs were identified based on 95 % HPD intervals and posterior probabilities (PPN0). Under the HPD approach, if zero was not included in the interval for the SNP effect, the SNP was declared as significant. In addition, if the PPN0 value was greater than 0.95, the SNP was also reported as significant.

From the significant SNPs, we identified one QTL region of four SNPs on chromosome 1 for SB and nine QTL regions for NT, with three on chromosome 7 (four, seven, and six significant SNPs in each QTL, respectively), five on chromosome 8 (six, four, five, four, and five significant SNPs in each QTL region, respectively) and one on chromosome 12, with four SNPs [See Additional file 9: Figure S7 and Additional file 10: Figure S8). In addition, 14 significant SNPs were identified for SB and 20 for NT that were not linked to other significant SNPs. Based on these QTL regions plus single SNP locations, we identified 18 and 57 genes that overlapped with significant SNPs, for SB and NT, respectively (Tables 1, 2).

**Table 1:** Significant SNPs for stillbirth and associated genes

SNP	Chr	Position (bp)	QTL	Gene	Distance (bp)^a^
BGIS0003207	1	183,948,686	–	*FEM1B*	14,336
				*PIAS1*	Inside
				*ITGA11*	6384
MARC0007670	1	193,689,396	–	–	–
M1GA0001259	1	204,647,860	QTL 1	*SAMD4A*	6863
ALGA0007251	1	204,871,282		*LOC102168145*, *WDHD1*, *SOCS4*, *MAPK1IP1L*, *LGALS3* and *DLGAP5*	Inside
MARC0056056	1	204,934,912			
H3GA0003422	1	205,020,361			
ASGA0010665	2	87,274,373	–	*F2R*	5412
				*LOC102165085/IQGAP2-like*	30,878
ALGA0014165	2	87,624,560	–	*LOC100520366*	Inside
MARC0000488	2	87,728,463	–	–	–
ALGA0014249	2	88,859,718	–	–	–
ALGA0018674	3	47,957,752	–	*NPHP1*	Inside
				*LOC102166895*	9013
ASGA0028841	6	82,315,974	–	*PTP4A2*	19,505
ASGA0070042	15	90,388,740	–	–	–
ALGA0086491	15	111,088,143	–	*LOC100738946/HECW2*	Inside
ASGA0070213	15	111,116,466	–	*LOC100738946/HECW2*	Inside
ASGA0072736	16	26,077,922	–	–	–
H3GA0049633	17	61,860,123	–	*CYP24A1*	31,229
ASGA0077857	17	61,889,054	–	*CYP24A1*	2298

The table shows significant SNPs, their positions in base pairs (bp) on the swine chromosome (Chr), the QTL region, associated genes (genes located in a QTL region or in an interval of 32.5 Kbp around each QTL region or SNP), followed by their distance in base pairs of a single marker or in relation to the first or last SNP of the region

^a^Gene location in relation to the QTL region or to the single SNP

**Table 2:** Significant SNPs for number of teats and associated genes

SNP	Chr	Position (bp)	QTL	Gene	Distance (bp)^a^
ALGA0004864	1	99,713,078	–	–	–
ALGA0012925	2	34,084,545	–	–	–
ALGA0012930	2	34,177,927	–	–	–
ALGA0013045	2	40,181,443	–	*LOC102167107*	20,750
MARC0055904	2	40,328,584	–	*SLC17A6*	Inside
ASGA0032215	7	31,600,286	QTL 1	*KLHL31*	978
H3GA0020592	7	31,714,979		*LOC102166539*, *LOC102166618*, *GCLC*, *LOC102167236* and *LOC102167364*	Inside
MARC0010879	7	31,869,398			
MARC0098266	7	31,945,954		*KHDRBS2*	12,332
ALGA0039995	7	32,047,280	QTL 2	*KHDRBS2*	Inside
ALGA0040000	7	32,134,452			
ASGA0032254	7	32,166,462			
ASGA0032255	7	32,192,051			
MARC0043689	7	32,252,888			
INRA0024655	7	32,313,430			
ASGA0032266	7	32,543,114		–	–
ALGA0040040	7	32,915,748	–	*PRIM2*	Inside
ASGA0034811	7	91,149,363	–	–	–
H3GA0022644	7	102,901,720	–	*PTGR2*	27,013
MARC0038565	7	103,495,170	–	*VRTN*	28,094
				*SYNDIG1L*	5675
MARC0048752	7	103,789,642	QTL 3	*AREL1*	5892
M1GA0010654	7	103,796,933		*FCF1*, *YLPM1*, *PROX2*, *DLST* and *RPS6KL1*	Inside
ALGA0043962	7	103,816,521			
H3GA0022664	7	103,910,821			
ASGA0035527	7	103,933,199			
DIAS0001088	7	103,960,033			
M1GA0010658	7	103,999,954	–	*LOC102167367*	26,610
				*PGF*	Inside
ASGA0035536	7	104,108,293	–	*MLH3*	16,923
				*LOC102167860*	5573
				*ACYP1*	Inside
				*ZC2HC1C*	1044
				*NEK9*	11,806
ALGA0122954	7	104,598,913	–	*JDP2*	Inside
				*LOC100624918/FLVCR2*	8951
ASGA0035556	7	105,224,235	–	*TGFB3*	14,323
				*IFT43*	Inside
MARC0093074	8	50,223,543	QTL 1	*LOC102166479*, *C8H4orf45* and *RAPGEF2*	Inside
H3GA0024861	8	50,329,649			
H3GA0024862	8	50,359,681			
H3GA0024868	8	50,479,231			
H3GA0052920	8	50,503,562			
ASGA0038804	8	50,537,893			
DRGA0008588	8	51,580,681	–	–	–
MARC0077695	8	53,929,233	–	–	–
ALGA0047895	8	55,647,917	QTL 2	*LOC102159670*	Inside
H3GA0024880	8	55,670,008			
H3GA0024879	8	55,749,069			
ALGA0047896	8	56,064,449			
ASGA0038818	8	56,175,366	–	–	–
H3GA0024884	8	56,642,918	QTL 3	–	–
ASGA0038820	8	56,673,496			
H3GA0024882	8	56,695,265			
ASGA0038822	8	56,764,454			
ALGA0047901	8	56,805,057			
MARC0013221	8	57,262,741	–	*LOC100519853*/*ZNF320*	Inside
				*LOC102160850*	692
INRA0029832	8	58,466,955	QTL 4	*LOC102165777*, *NMU*, *LOC102166140*, *PDCL2*, *LOC102166866*, *CLOCK* and *LOC100523623/TMEM165*	Inside
ALGA0047932	8	58,557,889			
ALGA0047933	8	58,625,849			
M1GA0011944	8	58,807,576		*SRD5A3*	13,606
MARC0000554	8	67,026,060	–	*LOC100737487/EPHA5-like*	Inside
ASGA0085207	8	69,065,977	QTL 5	–	–
MARC0020237	8	69,070,421			
MARC0095739	8	69,146,481			
ALGA0103392	8	69,146,919			
ALGA0102491	8	69,215,722			
ALGA0066725	12	50,281,949	QTL 1	*METTL16*, *PAFAH1B1*, *LOC102165360*, *CLUH*, *LOC102165602/CCDC92*	Inside
ASGA0054883	12	50,340,265			
MARC0027202	12	50,489,072			
ALGA0066740	12	50,578,018			
DIAS0001557	12	55,962,023	–	*PFAS*	11,951
				*RANGRF*	Inside
				*SLC25A35*	163
				*ARHGEF15*	8954
				*ODF4*	31,445
ASGA0062949	14	43,688,399	–	*TRPV4*	4852
				*FAM222A*	4716

The table shows significant SNPs, their positions in base pairs (bp) on the swine chromosome (Chr), the QTL region, associated genes (genes located in a QTL region or in an interval of 32.5 Kbp around each QTL region or SNP), followed by their distance in base pairs of a single marker or in relation to the first or last SNP of the region

^a^Gene location in relation to the QTL region or to the single SNP

### Gene-TF networks

**Fig. 2:**
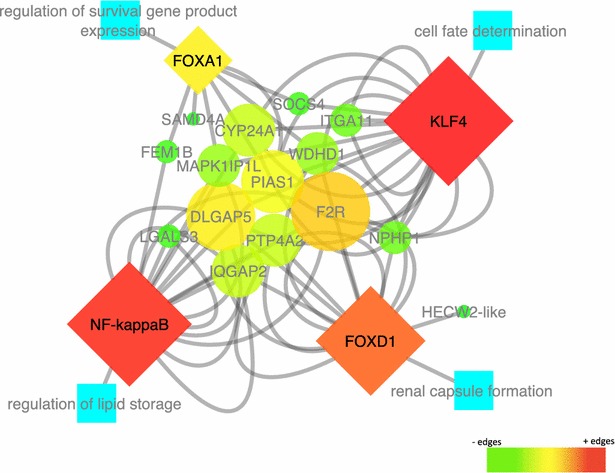
Stillbirth gene-transcription factor (TF) network. Four transcription factors associated with genes involved in stillbirth: *NF*-*κB*, *FOXA1*, *KLF4,* and *FOXD1* (*diamond nodes*), with in silico validated targets (*circle nodes*). Their color scale and size corresponds to network analyses (*Cytoscape*) scores, where *red* and *bigger nodes* represent higher edge densities, *while green* and *smaller nodes* represent lower edge densities. *Blue nodes* are the transcription factor related biological processes

**Fig. 3:**
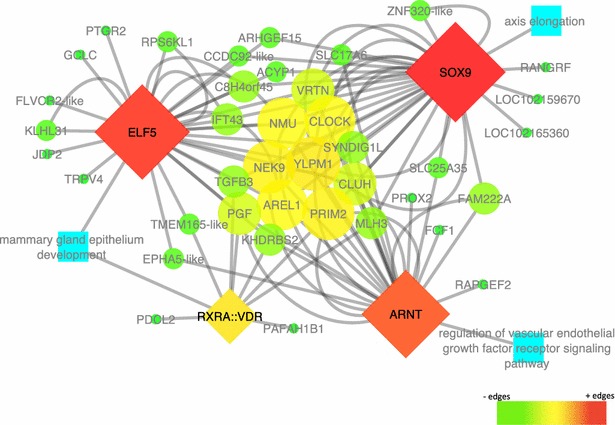
Number of teats gene-transcription factor network. Four transcription factors associated with genes involved in number of teats: *SOX9*, *ELF5*, *RXRA::VDR,* and *ARNT* (diamond nodes), with in silico validated target genes (*circle nodes*). Their color scale and size corresponds to network analyses (*Cytoscape*) scores, where *red* and *bigger nodes* represent higher edge densities, while *green* and *smaller nodes* represent lower edge densities. *Blue nodes* are the transcription factor related biological processes

Information about biological processes, cellular components, and molecular functions of all identified genes was based on human gene annotations, since they are more thorough and accurate than pig genome annotations [See Additional file 11: Table S3 and Additional file 12: Table S4]. Using the TFM-explorer, regulatory sequence analyses were performed to identify TF that were strongly related (p value <0.0001) to each set of genes for each trait [See Additional file 13: Table S5 and Additional file 14: Table S6]. The most representative TF genes (*FOXA1, FOXD1, NF*-*kappaB,* and *KLF4* for SB and *ARNT, ELF5, RXRA::VDR,* and *SOX9* for NT), according to the biological processes in which they are involved and to data in the literature, (e.g. TF involved with kidney diseases and diabetes for SB and TF related to mammary gland tissue and vertebrae composition and spinal cord expression for NT) were chosen to achieve a gene-TF network (Figs. 2, 3). Based on these networks, we were able to identify the most likely candidate genes for SB (*CYP24A1, DLGAP5, F2R, IQGAP2, LGALS3, MAPK1IP1L, NPHP1, PTP4A2, PIAS1,* and *WDHD1*) and NT (*PRIM2, AREL1, CLOCK, NEK9, NMU, SYNDIG1L, TGFB3, TMEM165*-*like,VRTN,* and *YLPM1*).

## Discussion

Based on DIC (Fig. 1), the Poisson model showed the best fit for SB, while the Gaussian model showed the best fit for NT. Most studies do not consider the Poisson distribution for SB [52], although the use of discrete distributions can lead to a more appropriate quantification of the genetic influences on this trait. Working under a hierarchical Bayesian approach, Varona and Sorensen [53] demonstrated the importance of proposing and comparing discrete distributions for SB data in animal breeding.

Although NT is also characterized as a discrete counting variable, the Gaussian distribution fits the behavior of this trait better than the Poisson distribution. A possible reason is that the Poisson distribution is asymmetric and right skewed, and the symmetry of the Gaussian distribution was more consistent with the observed distribution of the NT sample data. Another explanation is that the Poisson distribution assumes that the mean is equal to its variance, a condition that may not have been met when working with NT, even when assuming an extra residual term (Model 2). In addition, the mean of NT (15.3) was much larger than the mean of SB (1.2), and the Gaussian distribution is a reasonable approximation for the Poisson distribution when the mean is greater than 10. In future studies, it would be interesting to consider other distributions for discrete random variables for which such a constraint (mean equal to variance) is not required, e.g. the negative binomial and generalized Poisson distributions, as proposed by Varona and Sorensen [53].

The GWAS for SB identified 18 significant SNPs that were on chromosomes 1, 2, 3, 6, 15, 16, and 17, along with one QTL region on chromosome 1 (Table 1). Several QTL for SB were previously reported near the chromosomal regions that were identified in this study for chromosomes 1, 6, 16, and 17 [2]. For NT, the GWAS identified 65 significant SNPs on chromosomes 1, 2, 6, 9, 11, and 14, along with QTL regions identified on chromosome 7, 8, and 12 (Table 2). Several previously reported QTL for NT were located in the same chromosomal regions in which SNPs and QTL were identified in this study [38, 54–57]. Identification of these SNPs that were associated with SB and NT in the GWAS and the replication of QTL that were identified in other studies gave us more confidence to evaluate the SNP effects and the biological function of their related genes when using post-GWAS analyses, such as gene-TF network analyses, which allowed us to propose a link between the traits, QTL, and overlapping genes.

### Gene-TF networks

Based on single SNPs and QTL regions, we identified 18 and 57 genes, for SB and NT, respectively. From these genes, we collected information about their GO terms and pathways (e.g. renal system and reproductive biological processes for SB genes and glandular epithelial cell and mammary gland development biological processes for NT genes). The promoter regions that were enriched in the most relevant TF for each trait based on the biological process in which they were involved and on data from the literature, were investigated for each set of genes for each trait to generate gene-TF networks that highlighted the most likely candidate genes for SB and NT.

### Stillbirth

Three of the four TF found for the SB gene-TF network (*NF*-*κB, FOXA1,* and *FOXD1*) have been reported to be involved in (human) kidney disease and diabetes [58–60]. These maternal diseases are associated with an elevated risk of stillbirth in humans [3–5]. The fourth TF found for the SB gene-TF network (*KLF4*) is related to vascular and cardiac disease [61]. Heart disease is one of the major causes of mortality and morbidity in the human perinatal period [5]. Among pregnant diabetic women, fetal hypoxia and cardiac dysfunction secondary to poor glycemic control are suggested as important pathogenic factors in stillbirths [4].

With these TF, we constructed a network that identified new candidate genes for SB (*F2R, IQGAP2, PTP4A2, WDHD1, PIAS1, DLGAP5, MAPK1IP1L, SOCS4, NPHP1,* and *CYP24A1*). *DLGAP5* was one of the most highly connected genes in the gene-TF network. Fragoso et al. [62] indicated that it is a strong predictor of adrenocortical tumors and it is known that adrenocortical functions are linked to renal diseases [63] that are associated with SB. *F2R* was another well-connected gene, which has a role in congenital heart disease [64]. In the network, these two genes were connected to each other through the *NF*-*κB, KLF4,* and *FOXD1* TF, which are related to kidney and heart diseases, thereby linking them with stillbirths.

In humans, a diabetic pregnancy affects the occurrence of stillbirths. In the SB network, *CYP24A1* was one of the well-connected genes and it has been shown to be significantly more expressed in placental tissue from women with gestational diabetes mellitus [65]. Other genes in the SB network related to diabetes are *PTP4A2, IQGAP2,* and *SOCS4* [66–68]. *Nephronophthisis 1* (*NPHP1*) was another strong candidate gene that was identified and is associated with juvenile nephronophthisis and Joubert Syndrome (JS) [69]. One of the key symptoms of JS is breathing abnormality during the neonatal period [70], which could also be linked to the occurrence of SB. Using this gene-TF network, we were able to confirm genes that are linked to SB, not only through their position at a QTL, but also through a known biological role.

### Number of teats

The TF *SOX9* and *ARNT* from the NT gene-TF network are mainly involved with vertebrae composition and spinal cord expression. For example, *SOX9* has been shown to be involved with campomelic dysplasia [71], a syndrome which, among other symptoms, is characterized by vertebrae malformation and a smaller number of ribs [72]. *ARNT*, a gene that encodes an aryl hydrocarbon nuclear regulatory factor and is expressed in the spinal cord during mouse development [73], and *ELF5* and *VDR* that are involved with mammary gland tissue development [74, 75] were detected in the GO analyses based on their association with biological processes of the mammary gland development (see the gene-TF network in Fig. 3).

With these TF, we constructed a network that identified new candidate genes for NT (*PRIM2, AREL1, YLPM1, NEK9, NMU, CLOCK, SYNDIG1L, TMEM165*-*like, TGFB3,* and *VRTN*). Among these genes, *YLPM1* was one of the well connected genes in the network and plays a role in the reduction of telomerase activity during differentiation of embryonic stem cells [76]. Another well connected gene in this network is *PRIM2*, which encodes a DNA primase (large subunit) and contains a significant SNP that was found to be associated with body length in a LW × Minzhu pig population [77]. Linked with this group of genes, we found genes that are related to the number of vertebrae such as *PROX2, VRTN,* and *SYNDIG1L* [38, 78], which may be related with the NT in pigs [8, 31]. The chromosomal regions that contain each of these genes have been explored in detail because they are significantly associated with NT [31, 38]. Other well connected genes in the gene-TF network are *NMU*, which is related to bone formation [79], and *TGFB3*, which is significantly associated with ossification of the posterior longitudinal ligament of the spine in humans [80]. *TMEM165*-*like* that is connected with two TF is associated with the mammary gland epithelium development GO term, and has been reported to be linked to developing, lactating, and involuting mammary gland [81]. These genes, as well as *AREL1, NEK9* and *CLOCK* that have been studied in detail at the molecular level were highlighted in the network as the most likely candidate genes for NT.

## Conclusions

We showed that the Poisson distribution best fitted the data for number of SB, whereas the Gaussian distribution was superior for NT in pig. To determine, which distribution is best for count traits in GWAS, both the Poisson and Gaussian distributions, together with other discrete distributions, should be tested. For both SB and NT, we observed associations between significant SNPs and genes that include these SNPs. The present study also provides information about these genes, thereby increasing our understanding of the molecular mechanisms that underlie SB and NT. In addition, we predicted gene interactions that were consistent with known newborn survival traits and mammary gland biology in mammals, and that led to the identification of candidate genes for SB (e.g. *DLGAP5, PTP4A2, IQGAP2, SOCS4, CYP24A1, F2R,* and *NPHP1*) and NT (e.g. *YLPM1, PROX2, VRTN, SYNDIG1L, PRIM2, TMEM165*-*like, NMU,* and *TGFB3*). Our results highlighted TF that may have an important role for SB and NT (e.g. *NF*-*kappaB* and *KLF4* for SB and *SOX9* and *ELF5* for NT). Nevertheless, these are complex traits that are subject to the action of a large number of genes that are regulated by several TF, many of which have yet to be identified.

## Additional files

10.1186/s12711-016-0189-x Histograms and descriptive statistics for the p values of the Geweke convergence test for breeding values (a and b) and SNP effects (c and d), respectively for the traits SB and NT.
10.1186/s12711-016-0189-x Trace plots and empirical distributions for the two most significant SNPs for SB.
10.1186/s12711-016-0189-x Trace plots and empirical distributions for the two most significant SNPs for NT.
10.1186/s12711-016-0189-x Density of stillborn (SB) and number of teats (NT) data when considering the observed, Poisson and Gaussian curves.
10.1186/s12711-016-0189-x Significant SNPs, position, and chromosome (chr) location on the *S. scrofa* reference genome (10.2), posterior mean, posterior probability under H_0_ (PPN0), and 95 % HPD (Highest Posterior Density) interval limits for SB.
10.1186/s12711-016-0189-x Significant SNPs, position, and chromosome (chr) location on the *S. scrofa* reference genome (10.2), posterior mean, posterior probability under H_0_ (PPN0), and 95 % HPD (Highest Posterior Density) interval limits for NT.
10.1186/s12711-016-0189-x Graphical summary (Manhattan plot) of genome-wide association results for SB. The x axis represents the genome in physical order, while the y axis shows the posterior probability under H_0_ (PPN0) for all SNPs.
10.1186/s12711-016-0189-x Graphical summary (Manhattan plot) of genome-wide association results for NT. The x axis represents the genome in physical order, while the y axis shows the posterior probability under H_0_ (PPN0) for all SNPs.
10.1186/s12711-016-0189-x QTL region on chromosome 1 (Chr 1) that contains significant SNPs for SB. Solid lines mark the QTL region.
10.1186/s12711-016-0189-x QTL regions on chromosomes 7, 8, and 12 (Chr 7, Chr 8, and Chr 12 respectively) that contain significant SNPs for NB. Solid lines mark the QTL regions.
10.1186/s12711-016-0189-x Pathway and Gene Ontology terms (GO) of genes associated with significant SNPs identified for SB.
10.1186/s12711-016-0189-x Pathway and Gene Ontology terms (GO) of genes associated with significant SNPs identified for NT.
10.1186/s12711-016-0189-x Transcription factors (TF) identified for genes associated with SB, location in base pairs (bp) of the window sequence that carries binding sites, and p-value of the window confidence that was obtained from the TFM-explorer tool.
10.1186/s12711-016-0189-x Transcription factors (TF) identified for genes associated with NT, location in base pairs (bp) of the window sequence that carries binding sites, and p-value of the window confidence that was obtained from the TFM-explorer tool.
