# Supplementary figures and images for: Revealing new candidate genes for reproductive traits in pigs: combining Bayesian GWAS and functional pathways
Source: Genet Sel Evol. 2016 Feb 1;48:9. doi: 10.1186/s12711-016-0189-x (PMC4736284; doi:10.1186/s12711-016-0189-x)

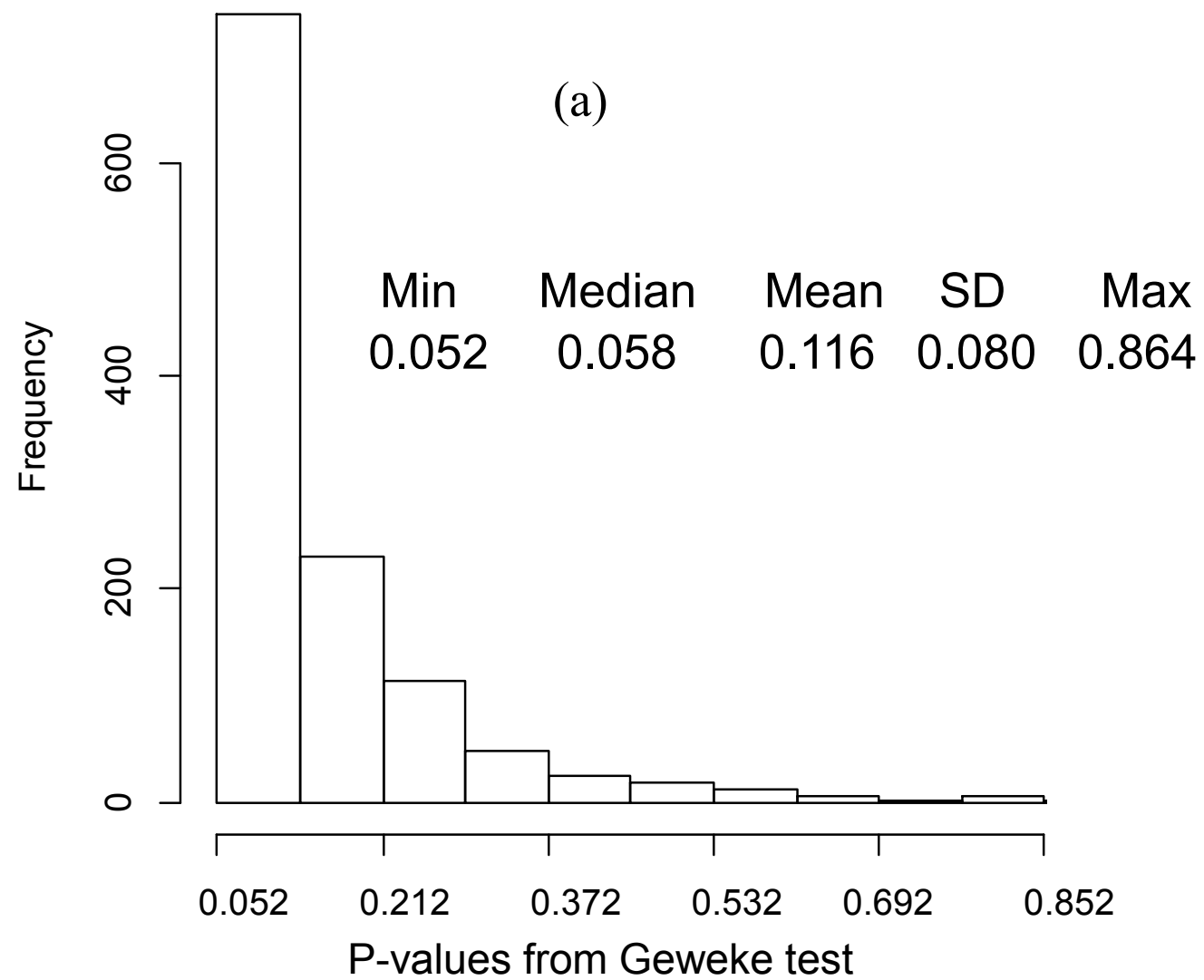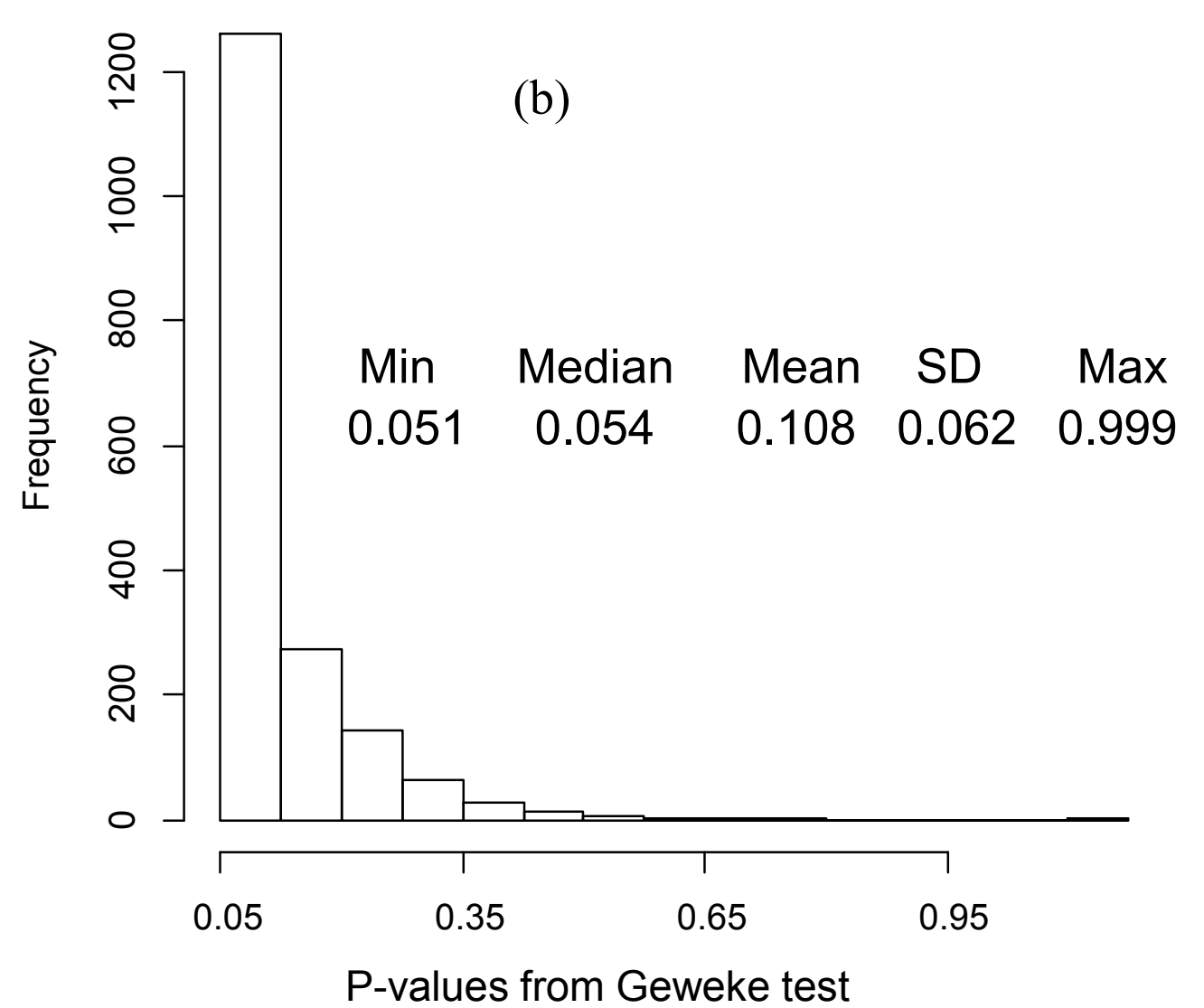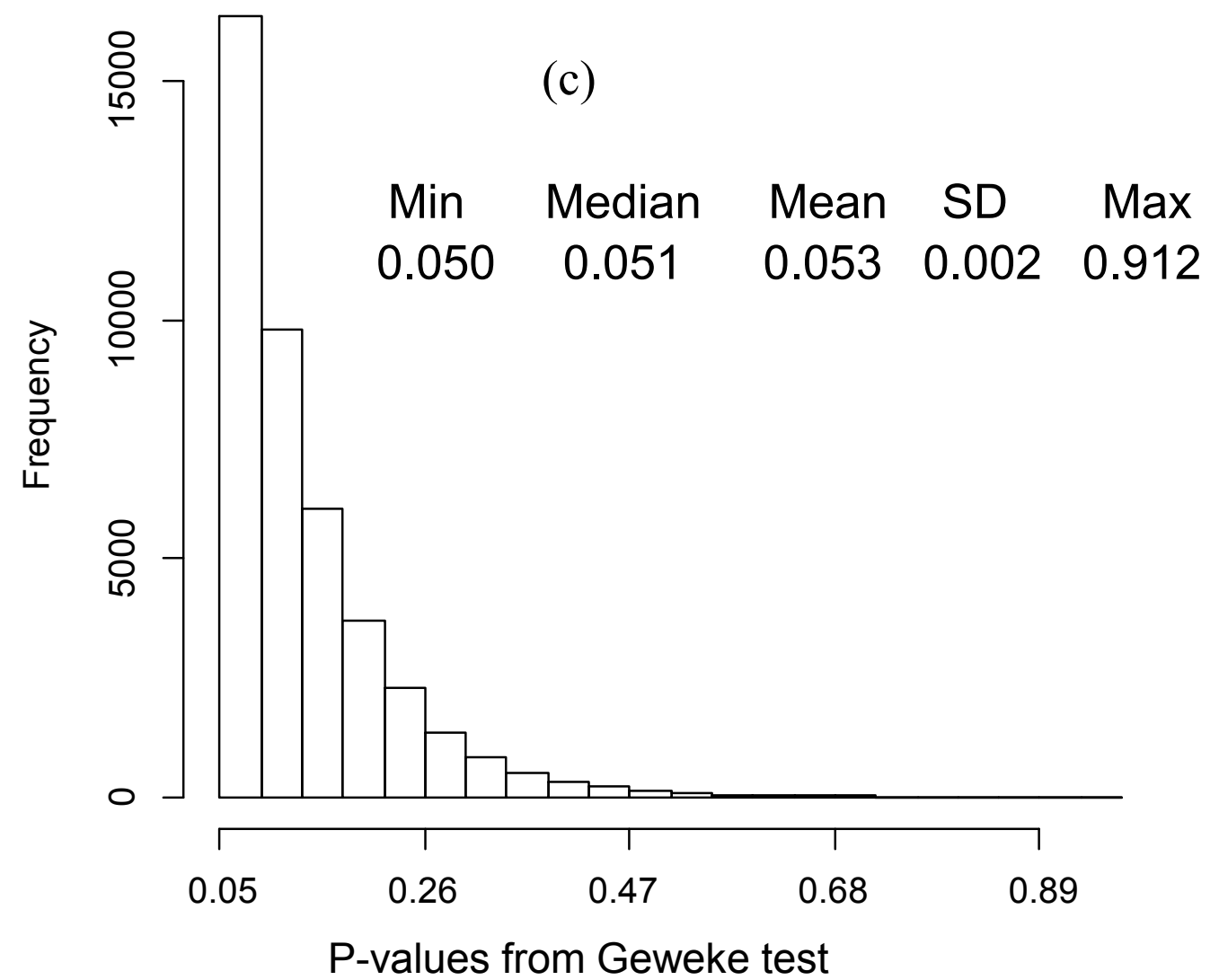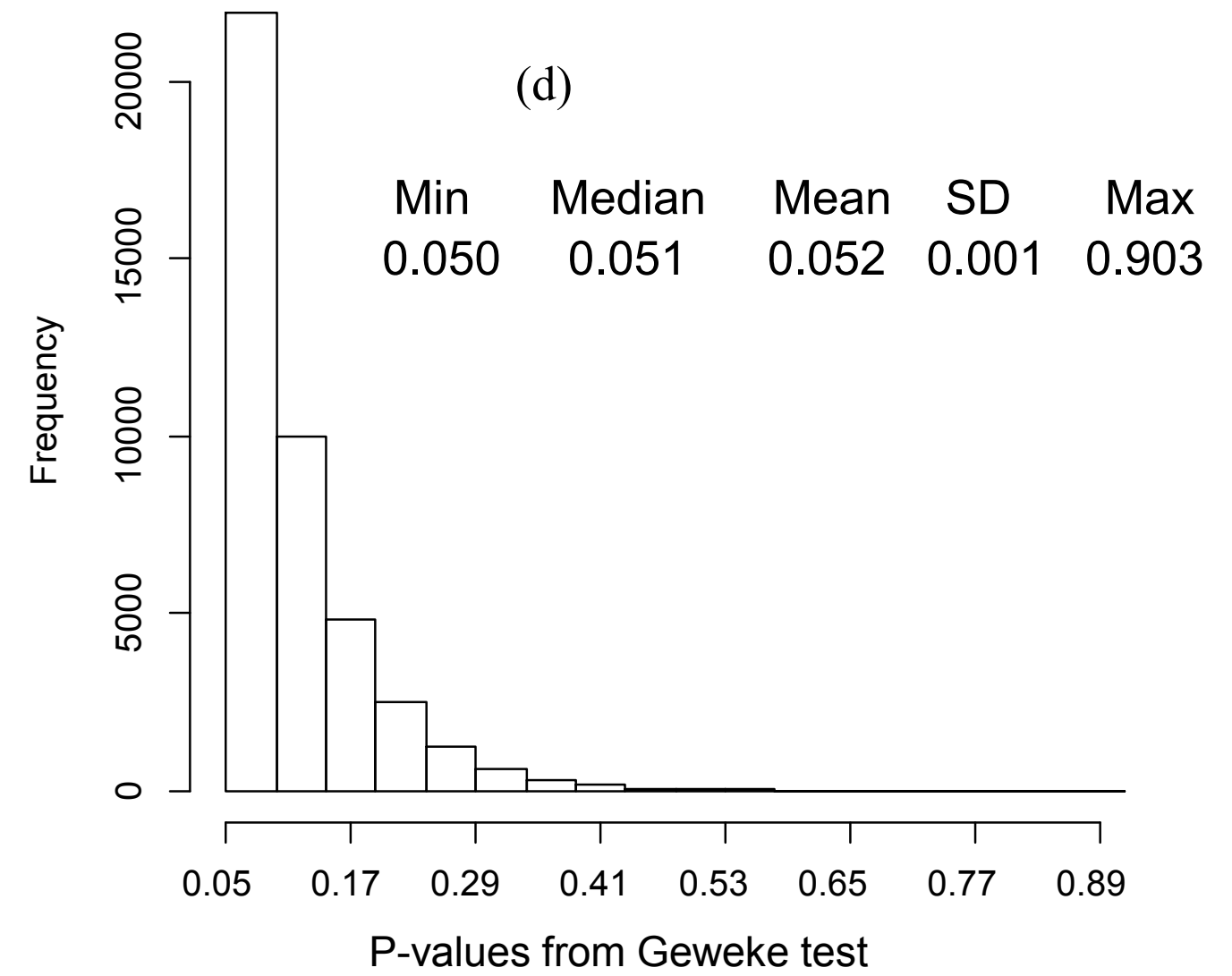

Supplement: Supplementary file 1 — 10.1186/s12711-016-0189-x Histograms and descriptive statistics for the p values of the Geweke convergence test for breeding values (a and b) and SNP effects (c and d), respectively for the traits SB and NT. [file 12711_2016_189_MOESM1_ESM.pdf]

# MARC0000488 , chr 2, pos 87728463 bp

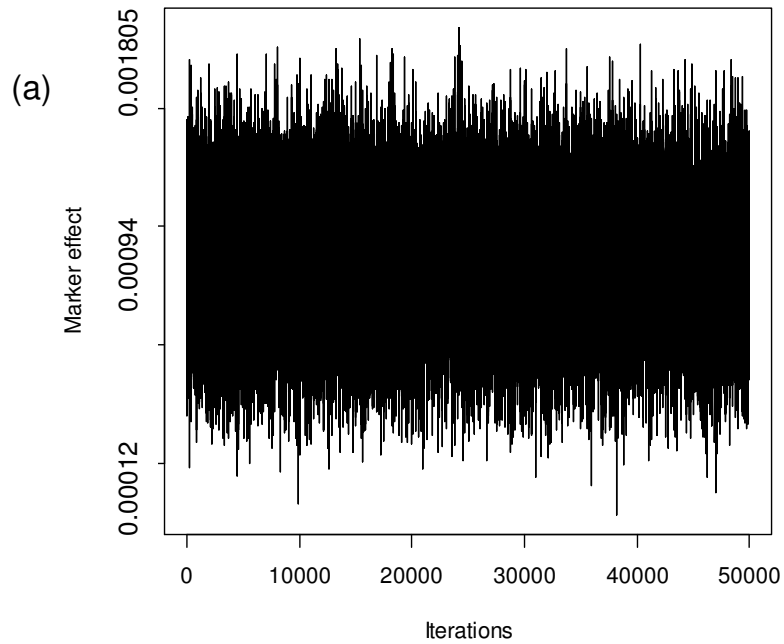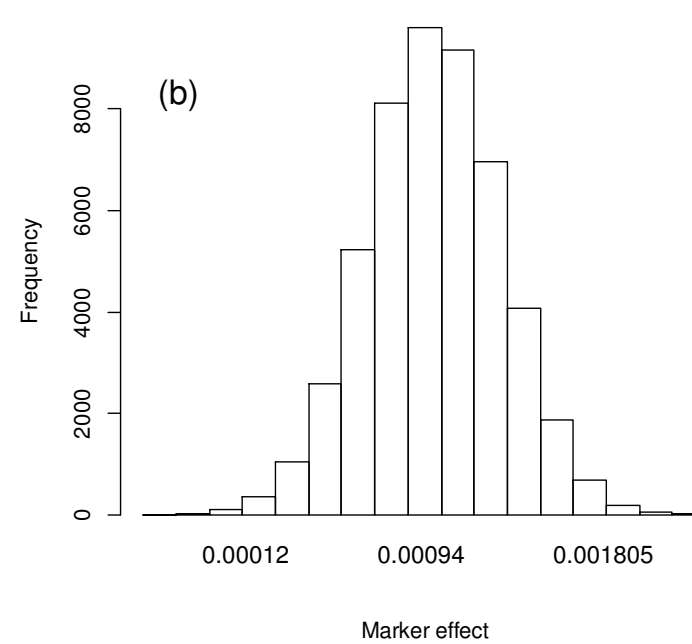

# ALGA0014165, chr 2, pos 87624560 bp

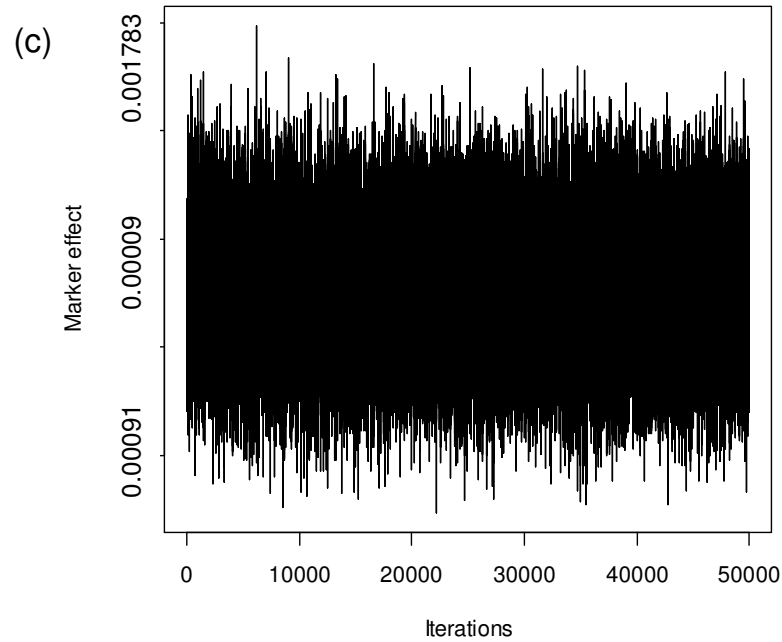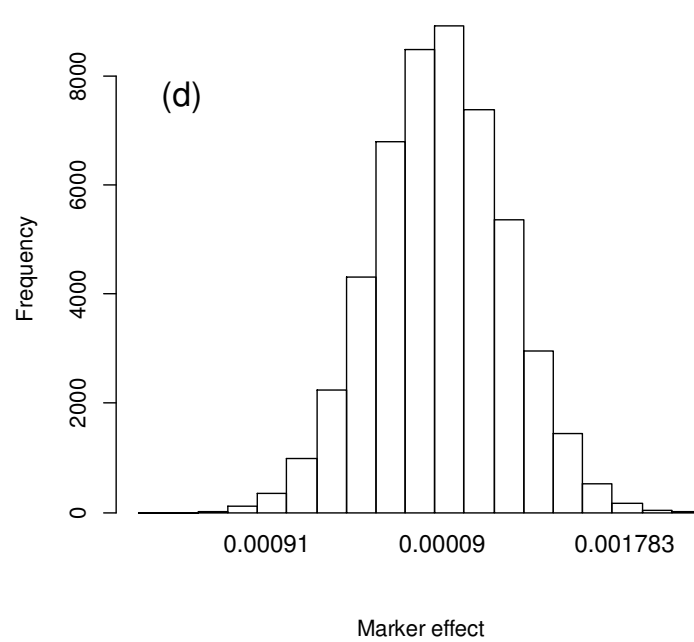

Supplement: Supplementary file 2 — 10.1186/s12711-016-0189-x Trace plots and empirical distributions for the two most significant SNPs for SB. [file 12711_2016_189_MOESM2_ESM.pdf]

### ALGA0004864, chr 1, pos 99713078 bp

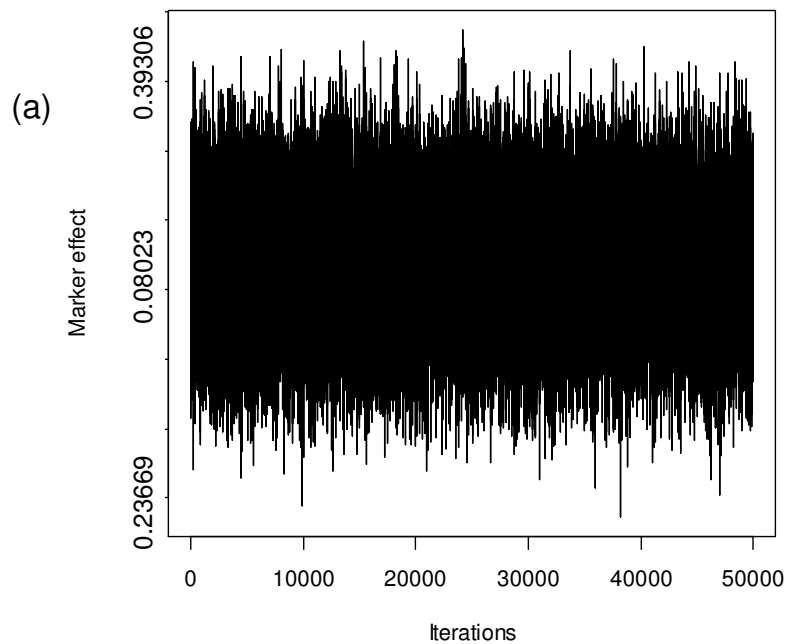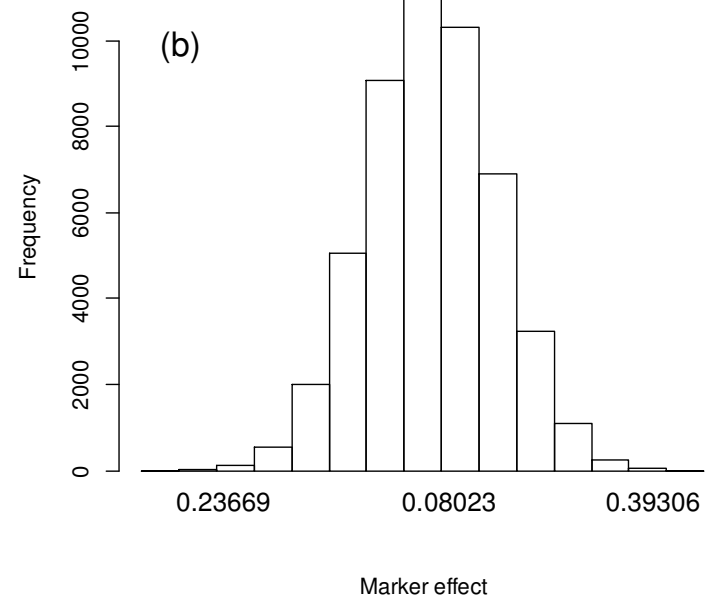

### ALGA0012930, chr 2, pos 34177927 bp

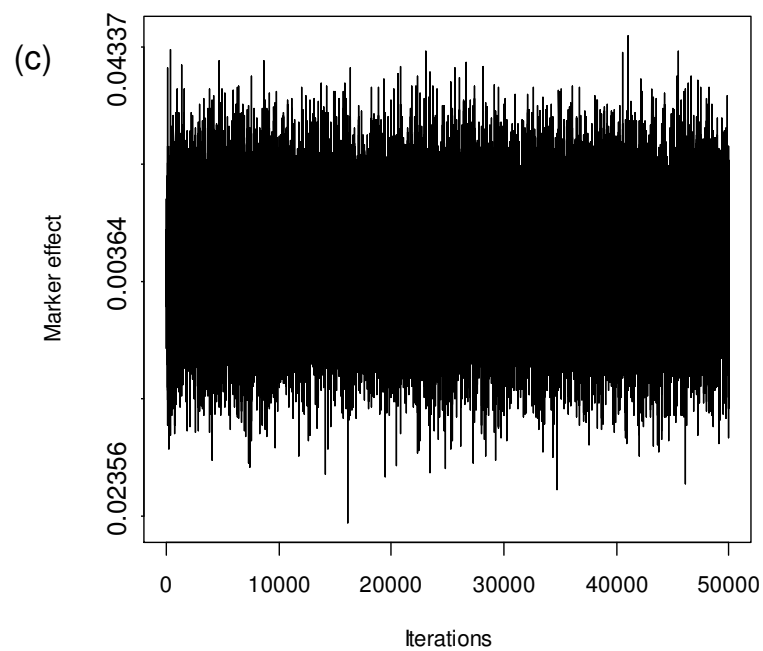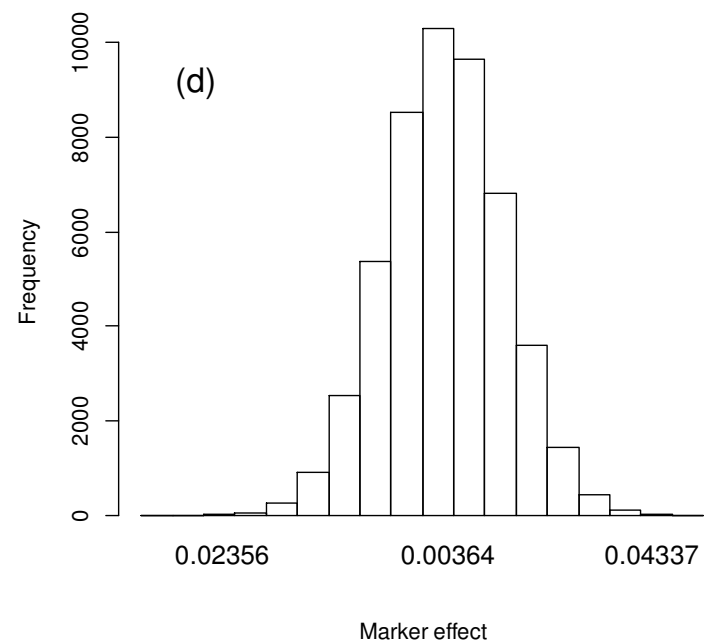

Supplement: Supplementary file 3 — 10.1186/s12711-016-0189-x Trace plots and empirical distributions for the two most significant SNPs for NT. [file 12711_2016_189_MOESM3_ESM.pdf]

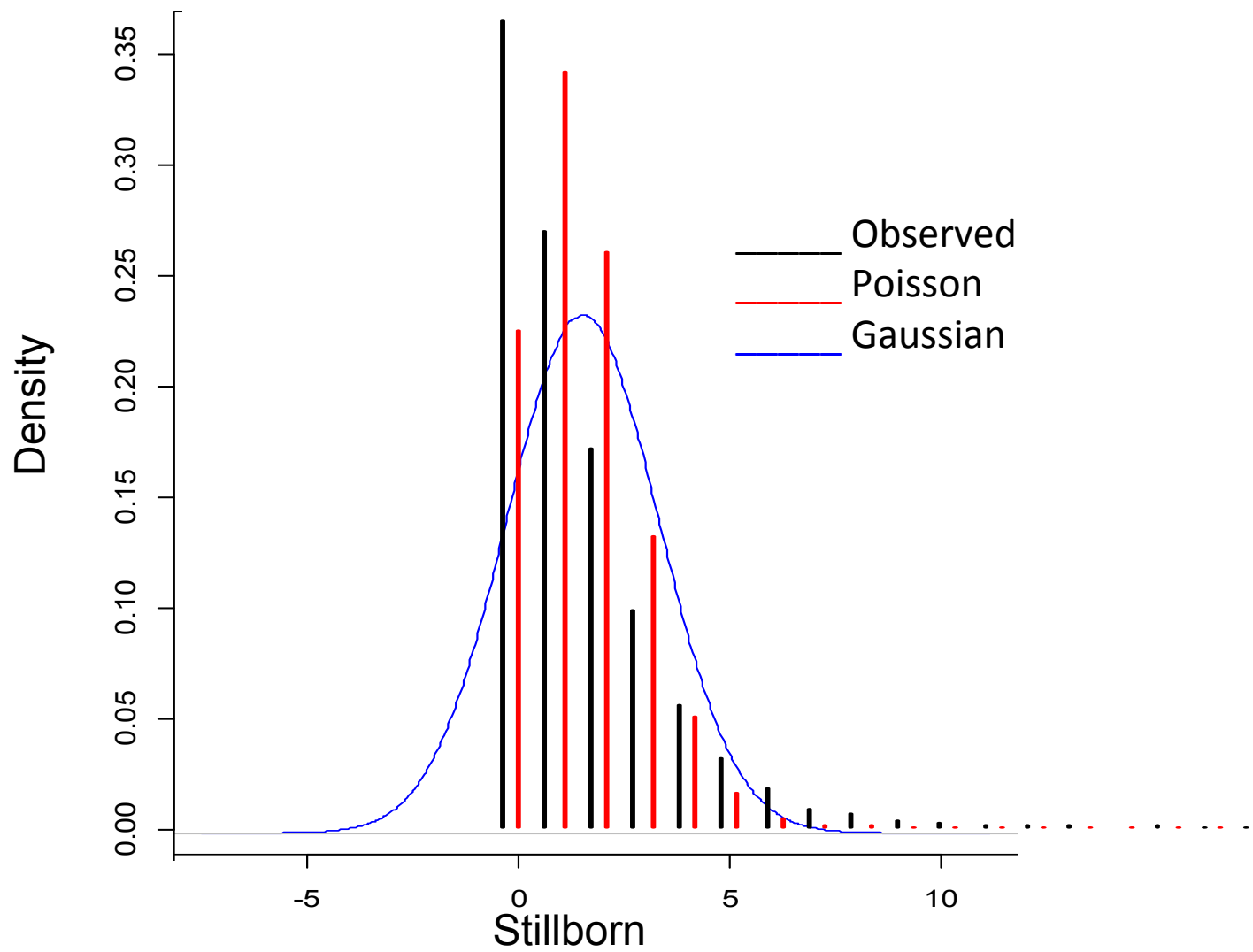

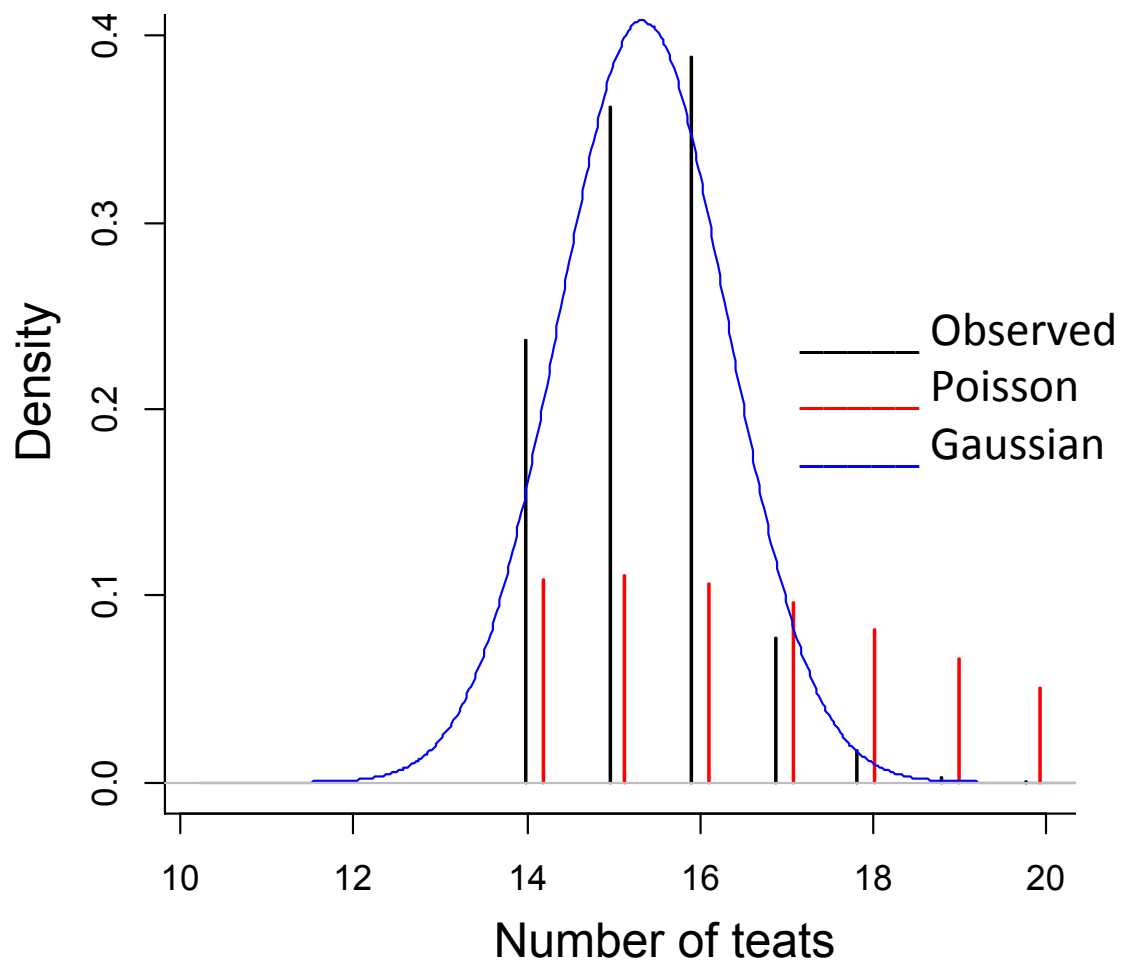

b

Supplement: Supplementary file 4 — 10.1186/s12711-016-0189-x Density of stillborn (SB) and number of teats (NT) data when considering the observed, Poisson and Gaussian curves. [file 12711_2016_189_MOESM4_ESM.pdf]

SB

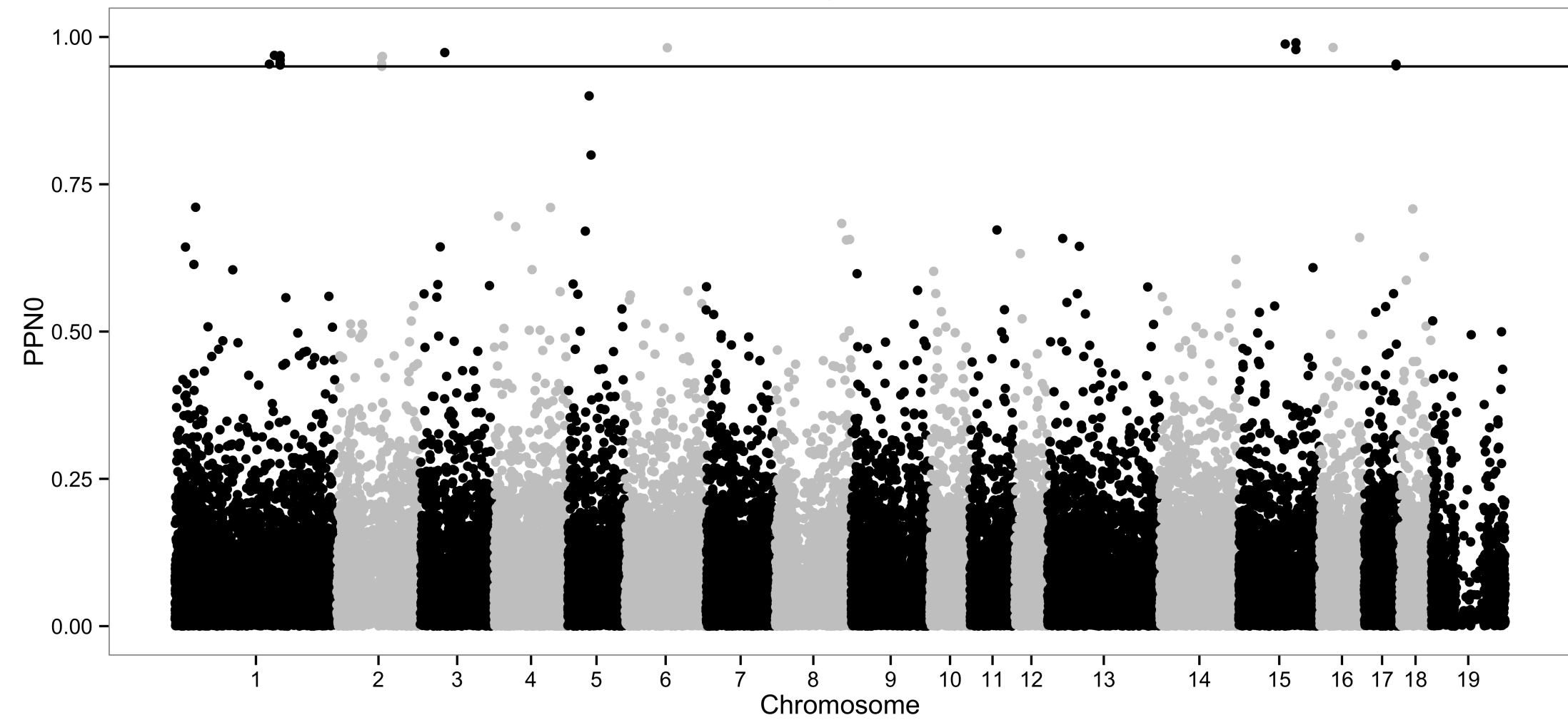

Supplement: Supplementary file 7 — 10.1186/s12711-016-0189-x Graphical summary (Manhattan plot) of genome-wide association results for SB. The x axis represents the genome in physical order, while the y axis shows the posterior probability under H0 (PPN0) for all SNPs. [file 12711_2016_189_MOESM7_ESM.pdf]

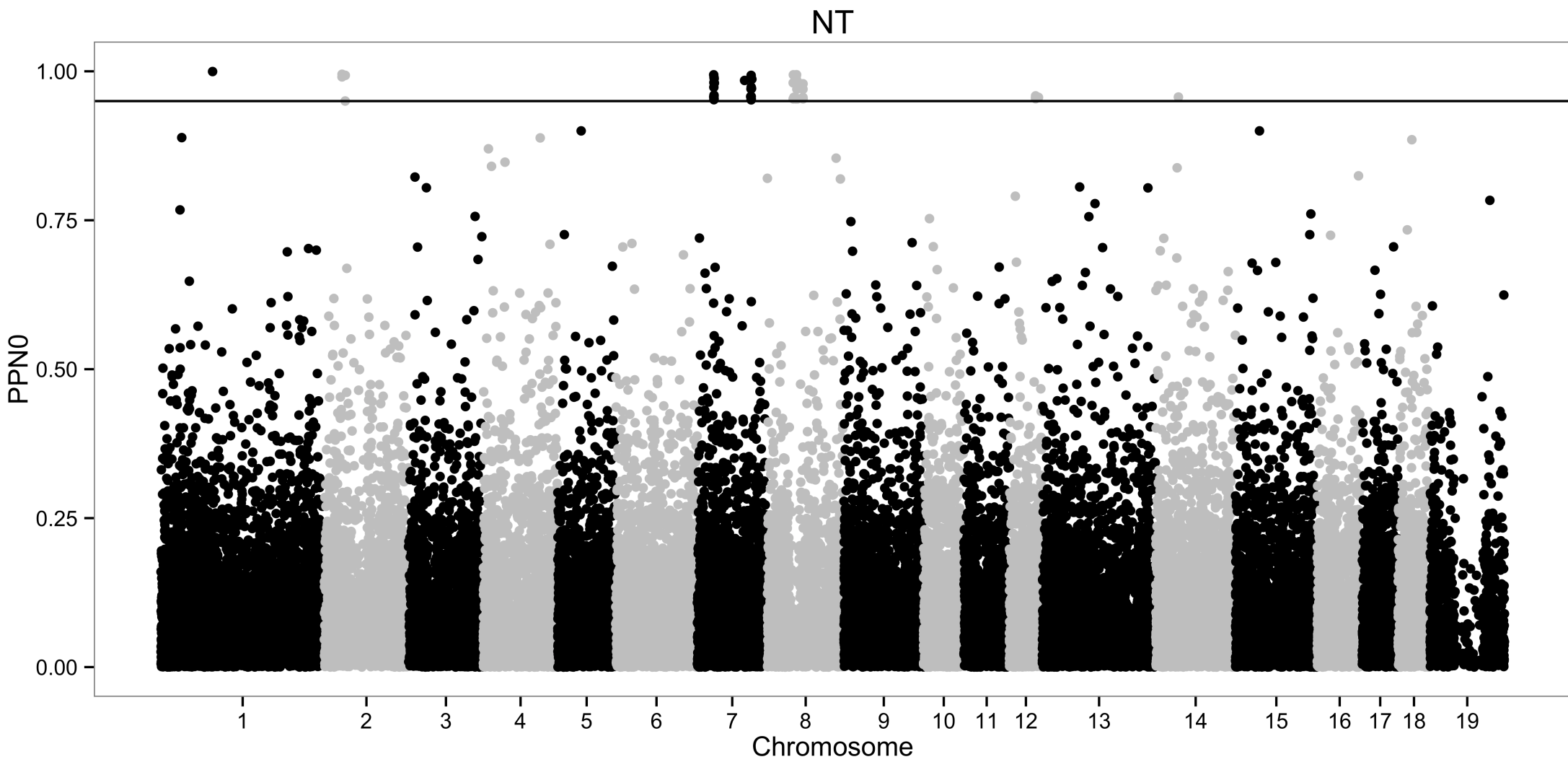

Supplement: Supplementary file 8 — 10.1186/s12711-016-0189-x Graphical summary (Manhattan plot) of genome-wide association results for NT. The x axis represents the genome in physical order, while the y axis shows the posterior probability under H0 (PPN0) for all SNPs. [file 12711_2016_189_MOESM8_ESM.pdf]

Chr 1

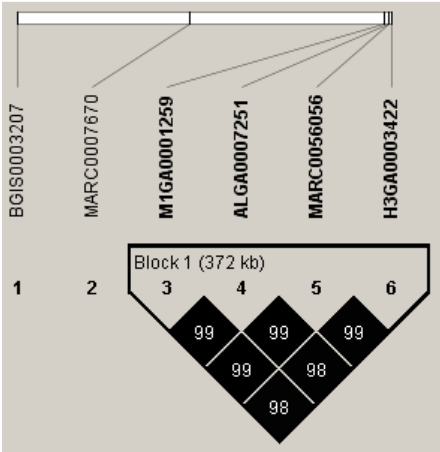

Supplement: Supplementary file 9 — 10.1186/s12711-016-0189-x QTL region on chromosome 1 (Chr 1) that contains significant SNPs for SB. Solid lines mark the QTL region. [file 12711_2016_189_MOESM9_ESM.pdf]

Chr 7

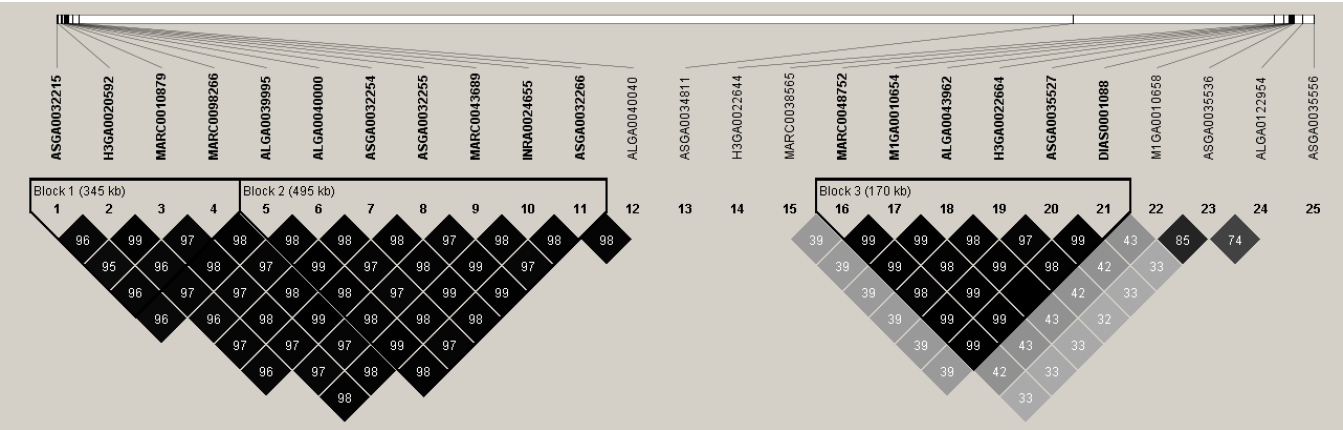

Chr 8

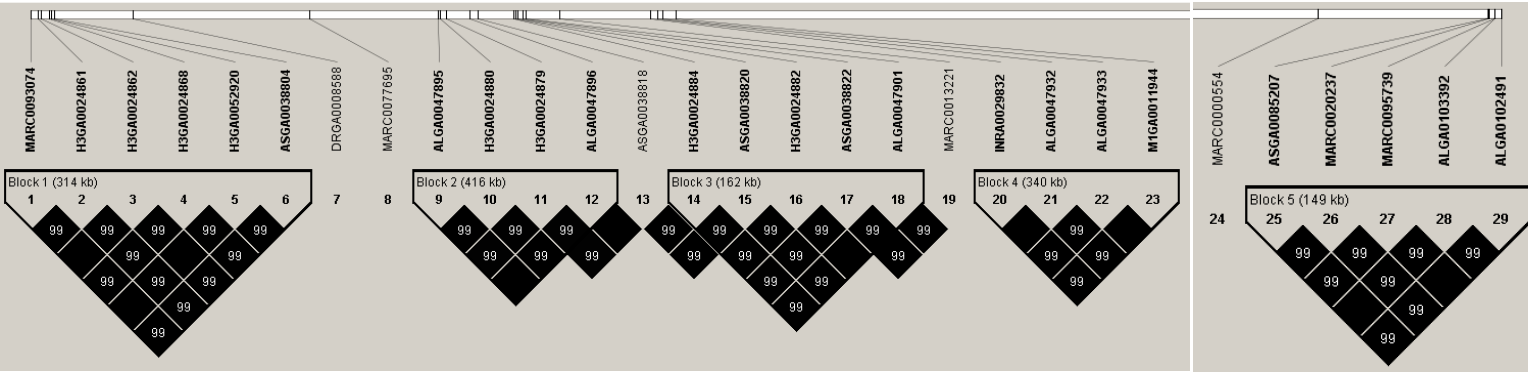

Chr 12

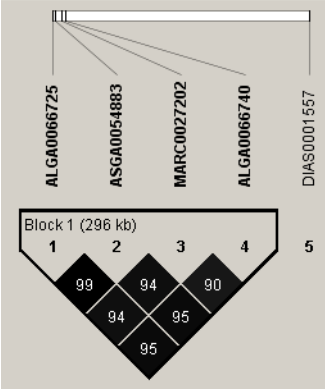

Supplement: Supplementary file 10 — 10.1186/s12711-016-0189-x QTL regions on chromosomes 7, 8, and 12 (Chr 7, Chr 8, and Chr 12 respectively) that contain significant SNPs for NB. Solid lines mark the QTL regions. [file 12711_2016_189_MOESM10_ESM.pdf]
