# Supplementary material for: Revealing new candidate genes for reproductive traits in pigs: combining Bayesian GWAS and functional pathways
Source: Genet Sel Evol. 2016 Feb 1;48:9. doi: 10.1186/s12711-016-0189-x (PMC4736284; doi:10.1186/s12711-016-0189-x)
Supplement: Supplementary file 5 — 10.1186/s12711-016-0189-x Significant SNPs, position, and chromosome (chr) location on the S. scrofa reference genome (10.2), posterior mean, posterior probability under H0 (PPN0), and 95 % HPD (Highest Posterior Density) interval limits for SB. [file 12711_2016_189_MOESM5_ESM.docx]

**Table S1** Significant SNPs, position, and chromosome (chr) location on *S. scrofa* reference genome (10.2), posterior mean, posterior probability under H_0_ (PPN0), and 95% HPD (Highest Posterior Density) interval limits for the stillborn trait.

|  |  |  |  |  | | **95% HPD interval** | | |
| --- | --- | --- | --- | --- | --- | --- | --- | --- |
| **SNP** | **chr** | **Position (bp)** | **mean** | **PPN0** | | **Lower** | **Upper** | |
| BGIS0003207 | 1 | 183948686 | 0.00100 | 0.96085 |  | 0.00003 | | 0.002009 |
| MARC0007670 | 1 | 193689396 | -0.00073 | 0.95015 |  | -0.00151 | | -0.000005 |
| M1GA0001259 | 1 | 204647860 | 0.00088 | 0.96699 |  | 0.00003 | | 0.001797 |
| ALGA0007251 | 1 | 204871282 | 0.00089 | 0.97354 |  | 0.00004 | | 0.001804 |
| MARC0056056 | 1 | 204934912 | 0.00089 | 0.97856 |  | 0.00005 | | 0.001796 |
| H3GA0003422 | 1 | 205020361 | 0.00086 | 0.95455 |  | 0.00001 | | 0.001769 |
| ASGA0010665 | 2 | 87274373 | 0.00087 | 0.98175 |  | 0.00007 | | 0.001716 |
| ALGA0014165 | 2 | 87624560 | 0.00091 | 0.98798 |  | 0.00009 | | 0.001783 |
| MARC0000488 | 2 | 87728463 | 0.00094 | 0.99014 |  | 0.00012 | | 0.001805 |
| ALGA0014249 | 2 | 88859718 | 0.00089 | 0.98213 |  | 0.00007 | | 0.001739 |
| ALGA0018674 | 3 | 47957752 | -0.00077 | 0.95105 |  | -0.00159 | | -0.000002 |
| ASGA0028841 | 6 | 82315974 | -0.00104 | 0.95389 |  | -0.00213 | | -0.000011 |
| ASGA0070042 | 15 | 90388740 | -0.00097 | 0.96842 |  | -0.00196 | | -0.000045 |
| ALGA0086491 | 15 | 111088143 | 0.00082 | 0.95388 |  | 0.00001 | | 0.001654 |
| ASGA0070213 | 15 | 111116466 | 0.00082 | 0.95455 |  | 0.00001 | | 0.001653 |
| ASGA0072736 | 16 | 26077922 | -0.00102 | 0.96587 |  | -0.00208 | | -0.000034 |
| H3GA0049633 | 17 | 61860123 | -0.00119 | 0.96875 |  | -0.00240 | | -0.000047 |
| ASGA0077857 | 17 | 61889054 | -0.00115 | 0.95241 |  | -0.00237 | | -0.000003 |
