# Supplementary material for: Revealing new candidate genes for reproductive traits in pigs: combining Bayesian GWAS and functional pathways
Source: Genet Sel Evol. 2016 Feb 1;48:9. doi: 10.1186/s12711-016-0189-x (PMC4736284; doi:10.1186/s12711-016-0189-x)
Supplement: Supplementary file 6 — 10.1186/s12711-016-0189-x Significant SNPs, position, and chromosome (chr) location on the S. scrofa reference genome (10.2), posterior mean, posterior probability under H0 (PPN0), and 95 % HPD (Highest Posterior Density) interval limits for NT. [file 12711_2016_189_MOESM6_ESM.docx]

**Table S2** Significant SNPs, position, and chromosome (chr) location on *S. scrofa* reference genome (10.2), posterior mean, posterior probability under H_0_ (PPN0), and 95% HPD (Highest Posterior Density) interval limits for the number of teats**.**

|  |  |  |  |  | **95% HPD interval** | |
| --- | --- | --- | --- | --- | --- | --- |
| **SNP** | **chr** | **Position (bp)** | **mean** | **PPN0** | **Lower** | **Upper** |
| ALGA0004864 | 1 | 99713078 | 0.23669 | 0.9996 | 0.08023 | 0.39306 |
| ALGA0012925 | 2 | 34084545 | -0.02302 | 0.99065 | -0.04290 | -0.00307 |
| ALGA0012930 | 2 | 34177927 | 0.02356 | 0.99500 | 0.00364 | 0.04337 |
| ALGA0013045 | 2 | 40181443 | 0.00120 | 0.95014 | 0.00001 | 0.00245 |
| MARC0055904 | 2 | 40328584 | 0.00121 | 0.95235 | 0.00001 | 0.00244 |
| ASGA0032215 | 7 | 31600286 | -0.00116 | 0.95403 | -0.00228 | -0.00009 |
| H3GA0020592 | 7 | 31714979 | 0.00115 | 0.98034 | 0.00007 | 0.00229 |
| MARC0010879 | 7 | 31869398 | 0.00121 | 0.99117 | 0.00013 | 0.00234 |
| MARC0098266 | 7 | 31945954 | -0.00108 | 0.95384 | -0.00213 | -0.00005 |
| ALGA0039995 | 7 | 32047280 | -0.00114 | 0.95391 | -0.00222 | -0.00010 |
| ALGA0040000 | 7 | 32134452 | -0.00121 | 0.95433 | -0.00230 | -0.00015 |
| ASGA0032254 | 7 | 32166462 | -0.00115 | 0.95399 | -0.00223 | -0.00009 |
| ASGA0032255 | 7 | 32192051 | -0.00112 | 0.95389 | -0.00217 | -0.00009 |
| MARC0043689 | 7 | 32252888 | -0.00124 | 0.95504 | -0.00244 | -0.00009 |
| INRA0024655 | 7 | 32313430 | -0.00119 | 0.95404 | -0.00227 | -0.00015 |
| ASGA0032266 | 7 | 32543114 | -0.00105 | 0.95383 | -0.00213 | -0.00001 |
| ALGA0040040 | 7 | 32915748 | -0.00112 | 0.95392 | -0.00222 | -0.00004 |
| ASGA0034811 | 7 | 91149363 | 0.00126 | 0.97123 | 0.00005 | 0.00251 |
| H3GA0022644 | 7 | 102901720 | 0.00246 | 0.99415 | 0.00051 | 0.00453 |
| MARC0038565 | 7 | 103495170 | -0.00239 | 0.99001 | -0.00454 | -0.00034 |
| MARC0048752 | 7 | 103789642 | 0.00186 | 0.95996 | 0.00002 | 0.00377 |
| M1GA0010654 | 7 | 103796933 | 0.00193 | 0.98797 | 0.00009 | 0.00384 |
| ALGA0043962 | 7 | 103816521 | 0.00191 | 0.98467 | 0.00008 | 0.00382 |
| H3GA0022664 | 7 | 103910821 | 0.00191 | 0.97346 | 0.00005 | 0.00383 |
| ASGA0035527 | 7 | 103933199 | 0.00188 | 0.97054 | 0.00004 | 0.00379 |
| DIAS0001088 | 7 | 103960033 | 0.00191 | 0.98677 | 0.00008 | 0.00383 |
| M1GA0010658 | 7 | 103999954 | 0.00211 | 0.99411 | 0.00049 | 0.00383 |
| ASGA0035536 | 7 | 104108293 | 0.00203 | 0.99222 | 0.00026 | 0.00390 |
| ALGA0122954 | 7 | 104598913 | 0.00208 | 0.99421 | 0.00050 | 0.00376 |
| ASGA0035556 | 7 | 105224235 | 0.00213 | 0.98544 | 0.00008 | 0.00431 |
| MARC0093074 | 8 | 50223543 | -0.00126 | 0.95612 | -0.00251 | -0.00007 |
| H3GA0024861 | 8 | 50329649 | -0.00130 | 0.95677 | -0.00255 | -0.00010 |
| H3GA0024862 | 8 | 50359681 | -0.00126 | 0.95616 | -0.00251 | -0.00006 |
| H3GA0024868 | 8 | 50479231 | -0.00129 | 0.95779 | -0.00258 | -0.00006 |
| H3GA0052920 | 8 | 50503562 | -0.00126 | 0.95622 | -0.00251 | -0.00007 |
| ASGA0038804 | 8 | 50537893 | -0.00128 | 0.95634 | -0.00253 | -0.00008 |
| DRGA0008588 | 8 | 51580681 | -0.00136 | 0.95714 | -0.00260 | -0.00017 |
| MARC0077695 | 8 | 53929233 | -0.00135 | 0.95765 | -0.00259 | -0.00017 |
| ALGA0047895 | 8 | 55647917 | -0.00105 | 0.95209 | -0.00208 | -0.00002 |
| H3GA0024880 | 8 | 55670008 | -0.00109 | 0.95384 | -0.00212 | -0.00006 |
| H3GA0024879 | 8 | 55749069 | -0.00112 | 0.95391 | -0.00223 | -0.00004 |
| ALGA0047896 | 8 | 56064449 | -0.00105 | 0.95376 | -0.00208 | -0.00002 |
| ASGA0038818 | 8 | 56175366 | -0.00105 | 0.95361 | -0.00208 | -0.00002 |
| H3GA0024884 | 8 | 56642918 | -0.00105 | 0.95371 | -0.00210 | -0.00001 |
| ASGA0038820 | 8 | 56673496 | -0.00107 | 0.95374 | -0.00211 | -0.00004 |
| H3GA0024882 | 8 | 56695265 | 0.00109 | 0.97938 | 0.00006 | 0.00213 |
| ASGA0038822 | 8 | 56764454 | -0.00104 | 0.95376 | -0.00208 | -0.00001 |
| ALGA0047901 | 8 | 56805057 | -0.00109 | 0.95386 | -0.00212 | -0.00006 |
| MARC0013221 | 8 | 57262741 | -0.00105 | 0.95372 | -0.00208 | -0.00002 |
| INRA0029832 | 8 | 58466955 | -0.00105 | 0.95369 | -0.00208 | -0.00002 |
| ALGA0047932 | 8 | 58557889 | -0.00105 | 0.95371 | -0.00208 | -0.00002 |
| ALGA0047933 | 8 | 58625849 | 0.00109 | 0.97808 | 0.00006 | 0.00213 |
| M1GA0011944 | 8 | 58807576 | 0.00109 | 0.97898 | 0.00006 | 0.00213 |
| MARC0000554 | 8 | 67026060 | 0.00098 | 0.97323 | 0.00005 | 0.00192 |
| ASGA0085207 | 8 | 69065977 | 0.00099 | 0.96997 | 0.00004 | 0.00195 |
| MARC0020237 | 8 | 69070421 | 0.00098 | 0.97004 | 0.00004 | 0.00193 |
| MARC0095739 | 8 | 69146481 | 0.00096 | 0.95875 | 0.00002 | 0.00191 |
| ALGA0103392 | 8 | 69146919 | 0.00099 | 0.97042 | 0.00004 | 0.00195 |
| ALGA0102491 | 8 | 69215722 | 0.00096 | 0.95903 | 0.00002 | 0.00191 |
| ALGA0066725 | 12 | 50281949 | 0.00209 | 0.99322 | 0.00031 | 0.00395 |
| ASGA0054883 | 12 | 50340265 | 0.00210 | 0.99324 | 0.00032 | 0.00396 |
| MARC0027202 | 12 | 50489072 | 0.00214 | 0.99398 | 0.00038 | 0.00397 |
| ALGA0066740 | 12 | 50578018 | 0.00185 | 0.98066 | 0.00007 | 0.00372 |
| DIAS0001557 | 12 | 55962023 | 0.00188 | 0.97901 | 0.00006 | 0.00380 |
| ASGA0062949 | 14 | 43688399 | 0.00143 | 0.98109 | 0.00007 | 0.00284 |
